# The origin of the parrotfish species *Scarus compressus* in the Tropical Eastern Pacific: region-wide hybridization between ancient species pairs

**DOI:** 10.1186/s12862-020-01731-3

**Published:** 2021-01-21

**Authors:** David B. Carlon, D. Ross Robertson, Robert L. Barron, John Howard Choat, David J. Anderson, Sonja A. Schwartz, Carlos A. Sánchez-Ortiz

**Affiliations:** 1 Schiller Coastal Studies Centre and Department of Biology, Bowdoin College, 6500 College Station, Brunswick, ME 04011 USA; 2 Smithsonian Tropical Research Institute, Balboa, Panama; 3 College of Science and Engineering, James Cook University, Townsville, QLD Australia; 4 Department of Environmental Science, Policy & Management, University of California, Berkeley, CA 94720 USA; 5 Departamento de Biología Marina, Universidad Autónoma de Baja California Sur, CP 23081 La Paz, Baja California Sur México

**Keywords:** Gene flow, Hybridisation, Introgression, Mating systems, Parrotfish, Postmating isolation, Premating isolation, Rocky reef, Speciation, Trimodal hybrid zone, Tropical Eastern Pacific

## Abstract

**Background:**

In the Tropical Eastern Pacific (TEP), four species of parrotfishes with complex phylogeographic histories co-occur in sympatry on rocky reefs from Baja California to Ecuador: *Scarus compressus*, *S. ghobban*, *S. perrico*, and *S. rubroviolaceus*. The most divergent, *S. perrico*, separated from a Central Indo-Pacific ancestor in the late Miocene (6.6 Ma). We tested the hypothesis that *S. compressus* was the result of ongoing hybridization among the other three species by sequencing four nuclear markers and a mitochondrial locus in samples spanning 2/3 of the latitudinal extent of the TEP.

**Results:**

A Structure model indicated that K = 3 fit the nuclear data and that *S. compressus* individuals had admixed genomes. Our data could correctly detect and assign pure adults and F1 hybrids with > 0.90 probability, and correct assignment of F2s was also high in some cases. NewHybrids models revealed that 89.8% (n = 59) of the *S. compressus* samples were F1 hybrids between either *S. perrico* × *S. ghobban* or *S. perrico* × *S. rubroviolaceus*. Similarly, the most recently diverged *S. ghobban* and *S. rubroviolaceus* were hybridizing in small numbers, with half of the admixed individuals assigned to F1 hybrids and the remainder likely > F1 hybrids. We observed strong mito-nuclear discordance in all hybrid pairs. Migrate models favored gene flow between *S. perrico* and *S. ghobban*, but not other species pairs.

**Conclusions:**

Mating between divergent species is giving rise to a region-wide, multispecies hybrid complex, characterized by a high frequency of parental and F1 genotypes but a low frequency of > F1 hybrids. Trimodal structure, and evidence for fertility of both male and female F1 hybrids, suggest that fitness declines sharply in later generation hybrids. In contrast, the hybrid population of the two more recently diverged species had similar frequencies of F1 and > F1 hybrids, suggesting accelerating post-mating incompatibility with time. Mitochondrial genotypes in hybrids suggest that indiscriminate mating by male *S. perrico* is driving pre-zygotic breakdown, which may reflect isolation of this endemic species for millions of years resulting in weak selection for conspecific mate recognition. Despite overlapping habitat use and high rates of hybridization, species boundaries are maintained by a combination of pre- and post-mating processes in this complex.

## Background

Hybridization has been documented in an increasing number of plant and animal systems [1] and has important consequences for evolutionary processes. Within populations or species, introgressive hybridization supplies new genetic variation over short evolutionary time scales that can provide the raw material for adaptation [2–7] and even rescue a species from severe environmental degradation [8]. At the macro-evolutionary level, hybridization and subsequent successful mating can result in transgressive segregation of unique traits [9, 10], producing novel phenotypes that may give rise to new species [11]. This process can spark adaptive radiation when highly divergent lineages hybridise and increase intraspecific variation in traits that facilitate diversification into vacant ecological niches [12, 13]. Alternatively, hybridisation can reverse diversifying processes that are important to adaptation and speciation. For example, when decreasing water clarity reduces the perception of visual mating cues in fishes, hybridization can weaken reproductive isolation and homogenize interspecific trait variation [14–16]. Understanding the ecological and genomic contexts that facilitate these divergent evolutionary scenarios calls for a broader systematic understanding of hybridization in the wild from a variety of empirical perspectives.

Fishes living on coral reefs are offering an increasing number of examples of variation in the dynamics of hybridisation, with an accelerating number of studies documenting hybrid zones in a diversity of evolutionary clades and biogeographical contexts [17–24]. The relatively high number of cases of hybridization documented across several ecologically important and diverse families of reef fishes, particularly the butterflyfishes (Chaetodontidae), angelfishes (Pomacanthidae), and wrasses (Labridae) [25], provide exciting opportunities to apply a comparative approach to hybridization and introgression in the wild, with the potential to illuminate the ecological, behavioural, and developmental mechanisms that facilitate or limit hybridization [19, 24, 26].

The Tropical Eastern Pacific (TEP) provides a unique biogeographical and ecological theatre to apply a comparative approach to hybridization in tropical marine fishes. The TEP is a relatively species-poor marine biogeographic region [27] that includes the coasts of Central and northern South America and the Galapagos and several other offshore islands. Patterns of species richness and the high level of regional endemism among its shorefish fauna (~ 80% [28]) have been heavily influenced by the isolation of the TEP from the rest of the Indo-Pacific by a 4000 km stretch of deep water known as the East Pacific Barrier (EPB), and the completion of the rise of the Isthmus of Panama (IOP) ~ 3 Ma [29]. The closure of the IOP reset faunas on both sides of the Isthmus and drove speciation and extinction in relation to the unique oceanographic features evolving on each side of the isthmus [30, 31]. In addition to endemic radiation within the tropical Americas, the TEP fauna also includes relatively recent colonists that have successfully crossed the EPB from the Central Pacific. For example, in both fishes [32] and corals [33], genetic data indicate that the TEP reef fauna includes colonists derived from Indo-Pacific lineages that arrived within the Pleistocene, potentially driven by favourable circulation patterns related to Plio-Pleistocene glaciation.

**Fig. 1:**
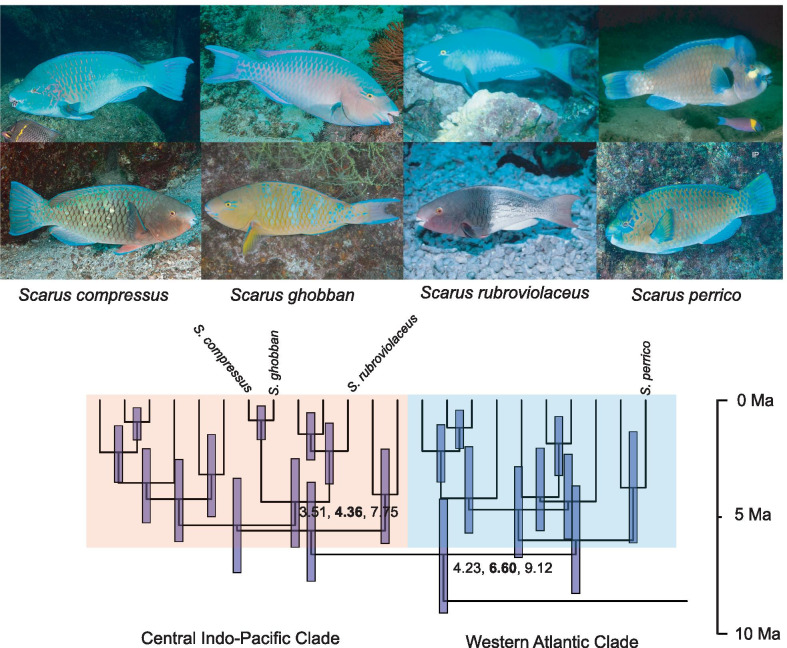
The evolutionary assembly of the *Scarus* species complex in the Tropical Eastern Pacific (TEP). Photographs illustrate phenotypic differences among species and colour phases. Top row: terminal phase fish (TP); Bottom row: initial phase fish (IP). Pruned species phylogeny and node ages (with 95% HPDs) adapted from [36] with permission. The two clades are colour coded by their most parsimonious biogeographic origin, see text for details. *Scarus perrico* and *S. compressus* are endemic to the TEP, while the other two species occur across the Indian and Pacific oceans. Photo credits: Andy Murch—IP and TP *S. ghobban*, IP and TP *S. compressus*, and IP phase of *S. perrico*. Kendall Clements—TP *S. perrico* and IP *S. rubroviolaceus*. D. Ross Robertson—TP *S. rubroviolaceus*

The parrotfishes (family—Labridae, tribe—Scarini) of the TEP include four sympatric *Scarus* species that co-occur on shallow (< 50 m) rocky reef habitats (hereafter, *Scarus* complex, Fig. 1). *Scarus perrico* (Jordan and Gilbert, 1882) is the sole surviving TEP representative of a Western Atlantic clade that split from an Indo-Pacific lineage by colonizing the proto-Caribbean Sea in the late Miocene (~ 6 Ma), and subsequently became subdivided between the Atlantic and Pacific by the rise of the Isthmus of Panama [34]. The sister lineage of *S. perrico* is *S. hoefleri* (Steindachner, 1881) which is restricted to West Africa [35]. The ancestor of the three other species in the *Scarus* TEP complex has a likely origin in the Central Indo-Pacific [36] (Fig. 1). This Central Indo-Pacific clade includes the widely distributed *Scarus rubroviolaceus* (Bleeker, 1849) and *S. ghobban* (Forsskål, 1775), which likely colonized the TEP from western source populations in a sequential fashion sometime during the last 0.35 Ma [32, 37]. The third TEP *Scarus* species in the Central Indo-Pacific clade: *S. compressus* (Osburn and Nichols, 1917) is endemic to the TEP and a phylogenetic hypothesis based on nuclear introns and mtDNA indicate it was recently derived from *S. ghobban* [35, 36].

Contrary to the sister species hypothesis for the relationship between *S. ghobban* and *S. compressus*, preliminary sequencing of a single copy nuclear region (*S7*) and a rapidly evolving mitochondrial marker (the mitochondrial control region—*mtCR*) suggests that the species *S. compressus* results from hybridization between two of the most divergent species pairs in the complex: *S. ghobban* and *S. perrico* (S. Schwartz, unpublished data). To rigorously test the hypothesis that hybridization among distantly related species could explain the origin and maintenance of *S. compressus*, we constructed a multi-locus genetic data set from samples collected at three localities that span much of the latitudinal range of the TEP, from the Gulf of California to 3700 km away in Panama (Additional file 1: Figure S1). To begin to identify the ecological and reproductive factors that promote hybridization in this complex, we simultaneously conducted ecological surveys of relative abundance of different species within each locality and collected complimentary morphological and reproductive data for each species. We use these data sets to confront the following six questions:

1. Does contemporary hybridization explain *Scarus compressus*?
2. What is the structure of the three hybrid zones with respect to F1 and > F1 hybrids, and does hybrid zone structure depend on the age of divergence between the parental species?
3. Is there evidence for historical gene flow and introgression among species pairs?
4. What is the relative abundance of the hybrid species *Scarus compressus*, and do the relative abundances of the hybrid varieties vary among localities?
5. Are male and female *S. compressus* fertile?
6. Is there diagnostic morphological and colour variation among species and hybrid varieties?

## Results

### Q1. Does contemporary hybridization explain *Scarus compressus?*

Contemporary hybridization between distantly related taxa will create unique signals in the mitochondrial and nuclear genomes of putative hybrids. For mitochondria, hybrids are expected to share genomes with one or both parents. For nuclear variation, first generation hybrids (F1) will be heterozygous for alleles that are common in both parents. Further, admixture approaches using nuclear data are expected to identify cases of mixed ancestry of hybrid samples, and the true number of randomly mating populations given a set of genotypic data [38]. We focused on the rapidly evolving mitochondrial control region (*mtCR*) and four more slowly evolving nuclear markers. Our nuclear markers target portions of two coding genes (*rag2* and *tmo4c4*) and two developmental regulatory regions (*dlx2* and *bmp4*). Rates of molecular evolution have been calibrated in *Scarus* for two of these markers, and range between 0.0102 and 0.0025 substitutions/million years [39]. These rates translate into diagnostic substitutions in lineages that are minimally several million years old and confident phasing of sanger sequencing reads at all four loci. At the same time, we recognize that modest sampling of the nuclear genome may not distinguish ongoing contemporary hybridization among three species from more complex hypotheses involving four species, very recent speciation, and abundant hybridization. We consider this hypothesis further in the “Discussion”.

#### Genetic variation within and among species

**Fig. 2:**
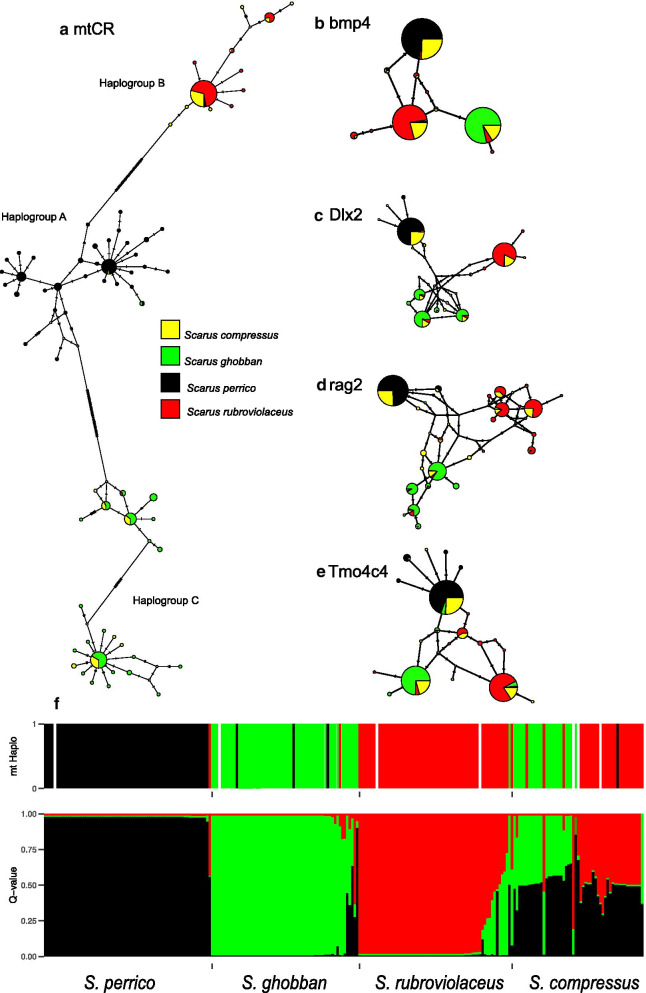
The distribution of mitochondrial and nuclear variation within and among species. **a**–**e** Haplotype networks for each of five genes with the frequency of each haplotype within species indicated by colour coding. **f** Bottom panel—Structure plot of K = 3 model based on the nuclear data (bottom panel). Top panel—mitochondrial haplogroup (defined in **a**) of each individual indicated by bar colour. Bars are colour coded to reflect haplogroup origin: black—*S. perrrico* (**A**) red—*S. rubroviolaceus* (**B**), green—*S. ghobban* (**C**). White bars indicate individuals in which *mtCR* was not sequenced

As expected from a hypothesis of hybrid origin, *Scarus compressus* shared common alleles with the three other species (Fig. 2a–e). The few cases of unique alleles sampled in *S. compressus* were all rare, and most were singletons. Summary statistics of mt and nuclear diversity show variation across localities within each of the four species. However, *S. compressus* had consistently high values of *S*, $\theta$, and $\pi$ regardless of gene and locality of collection, reflecting a high degree of divergence between mt genomes or maternal and paternal chromosomes (Additional file 2: Table S1). Further, tests of linkage disequilibrium (LD) within localities found the highest number of significant LD tests (11 of 14 possible tests) in *S. compressus*, consistent with the effects of hybrid zones on the probability of recombination among loci (Additional file 3: Table S2). Interestingly, the species with the second highest number of significant LD tests was *S. rubroviolaceus* (6/9 tests) which may also reflect its participation in hybridization with both *S. perrico* and S. *ghobban* and/or departures in non-random mating due to harem social organization and pair-spawning. In each of the three biological species, the overall patterns of significant LD tests between specific locus pairs vary among localities, suggesting demographic factors and not physical linkage among marker loci is the cause of significant LD in those cases.

#### Structure model, admixture, and mito-nuclear discordance

Evaluating Structure models of K ranging from 1 to 6 with the Delta K statistic strongly supports K = 3 as the best fitting model (Additional file 4: Table S3). Models of K > 3 resulted in low assignment values in clusters > 3, and no additional substructure was visible in the Structure plots. In the K = 3 model, individual samples from three species: *S. perrico, S. ghobban*, and *S. rubroviolaceus*; generally had high assignment values into one of the three clusters, while the majority of *S. compressus* individuals had admixed genomes (Fig. 2f). Admixed *S. compressus* could be further divided into two groups. Nearly all of the *S. compressus* samples had approximately 50% assignment in the *S. perrico* cluster, with the remaining assignment to either the *S. ghobban* or *S. rubroviolaceus* cluster (Fig. 2f lower panel). Nine phenotype samples of *S. rubroviolaceus* also had admixed genomes of varying Q-values with the *S. ghobban* cluster, suggesting hybridization between these two species.

Three divergent mitochondrial clades were identified from sequencing the *mtCR* gene, which we refer to as A, B, and C haplogroups (Fig. 2a). Each mt haplogroup was associated with one of the three, non-hybrid species (Fig. 2a). In individuals with admixed genomes, which included nearly all of the *S. compressus* samples and a smaller fraction of the *S. rubroviolaceus* samples, there were strong asymmetries in the species donor of the mitochondrial genome (Fig. 2f, Table 1). *Scarus compressus* individuals typically carried the haplogroups that matched the haplogroups of *S. ghobban* and *S. rubroviolaceus*. A similar pattern was found in *S. rubroviolaceus* phenotypes with admixed *S. ghobban* × *S. rubroviolaceus* nuclear backgrounds, in that only the *S. rubroviolaceus* B haplogroup was sampled in these individuals. We found evidence for mito-nuclear discordance in three *S. ghobban* individuals (n = 60), in each case a *S. perrico* mt genome (A haplogroup) was sampled in pure *S. ghobban* nuclear backgrounds (Fig. 2f).

**Table 1:** Tests of asymmetrical rate evolution in mt vs. nuclear genes in three hybrid crosses

Species pair		mt haplogroup (%)				${\delta }_{i}$		
a	b	A	B	C	Predicted asym	a	b	Species > mt evolution
*S. ghobban* (C)	*S. rubroviolaceus* (B)	0.00	84.6	15.4	a > b	0.109	− 0.109	*S. ghobban*
*S. perrico* (A)	*S. ghobban* (C)	0.00	7.1	92.9	a >> b	− 0.065	0.065	*S. ghobban*
*S. perrico* (A)	*S. rubroviolaceus* (B)	3.4	93.1	3.4	a >> b	0.051	− 0.051	*S. perrico*

Shaded columns indicate asymmetry that is consistent with mitonuclear DMIs

Species-specific mt haplogroups are given in parenthesis after each species in the species pair column and correspond to Fig. 2a. The “Predicted asym.” column gives the predicted direction of asymmetry between species given the frequency of mt haplogroups in hybrids (“mt haplogroup” column). The asymmetry parameter ${\delta }_{i}$ is calculated from estimates of mitochondrial and nuclear branch lengths on a phylogenetic hypothesis (Additional file 1: Figure S4). See methods for more details

The admixture results, combined with mt haplotyping, indicate that *S. compressus* phenotypes have the genomic signals of first-generation hybrids, the result of mating between either: *S. perrico* × *S. ghobban* or *S. perrico* × *S. rubroviolaceus.*

### Q2. What is the structure of the three hybrid zones with respect to F1 and > F1 hybrids, and does hybrid-zone structure depend on the age of divergence between the parental species?

#### Assignment to hybrid classes

Our nuclear genotypic data allows us to employ the NewHybrids approach [40] which uses a Bayesian model to determine the posterior probabilities of first generation hybrids (F1) and two hybrid classes consisting of deeper generational hybrids (F2 and backcrosses) given a sample of pure parents. Since the power of NewHybrids to detect and correctly assign individuals to the two deeper generation classes will depend on the number of loci, allelic diversity, and allele frequencies in each parental species, we also ran a series of complimentary power simulations using the Hybriddetective [41] workflow.

**Fig. 3:**
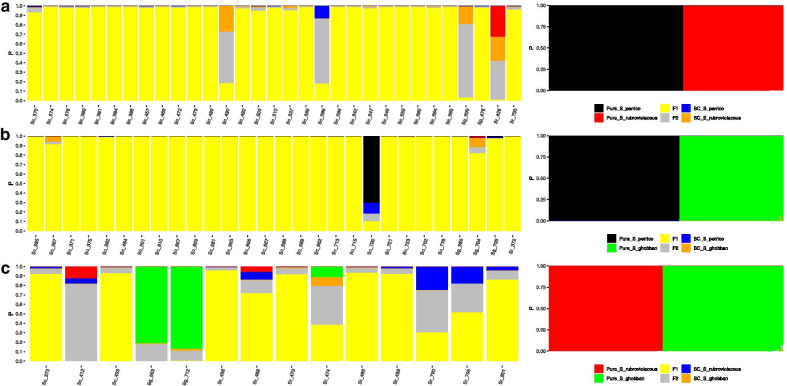
Probability of assignment of individuals into four hybrid classes based on three NewHybrids models. **a** *S. perrico* × *S. rubroviolaceus* cross, **b** *S. perrico* × *S. ghobban* cross, **c** *S. rubroviolaceus* × *S. ghobban* cross. Right panels are for two pure parental assignments, left panels are for the four hybrid classes, F1 hybrids, F2 hybrids, and the two possible backcrosses

Assignment with NewHybrids models revealed hybrid zones dominated by F1 classes for both *S. perrico* × *S. ghobban* data and the *S. perrico* × *S. rubroviolaceus* data using a critical posterior probability of 0.85 (Fig. 3). For the *S. perrico* × *S. ghobban* data, 93% of the hybrids (n = 28) were classified as F1, while the remaining two individuals had mixed assignments (Fig. 3b). For the *S. perrico* × *S. rubroviolaceus* data, 87% of the hybrids were classified as F1, while the remaining four individuals had mixed assignments (Fig. 3a). Notably, in the *S. perrico* × *S. rubroviolaceus* data, one of the mixed assignment individuals had an F2 assignment with a posterior probability of 0.78. In light of the results from our power analysis described in the next paragraph, we have high confidence that this individual is indeed an F2 hybrid. For the *S. ghobban* × *S. rubroviolaceus* crosses (Fig. 3c) a lower percentage of hybrids were assigned to the F1 class (50%, n = 14). Of the remaining seven individuals, two were assigned to the pure *S. ghobban* class (P > 0.92), four had mixed assignments, and one had high F2 assignment (P = 0.82). Given the high false positive rate for F2 individuals in the *S. ghobban* × *S. rubroviolaceus* power simulations, nothing more can be said with confidence about the five hybrid individuals not classified as F1, other than they can be assigned to a group that includes > F1 generation hybrids.

Our simulations revealed that the power to detect pure individuals and F1 hybrids across all three crosses with NewHybrids was high (> 0.90, Additional file 1: Figure S2a). In contrast, power to detect F2 hybrids and individuals that result from backcrosses was uniformly low (< 0.40). Breaking down the two components of power, for each cross revealed that accuracy (correct assignment) of F2 hybrids was high (> 0.90) at critical posterior thresholds approaching 0.80 or more (Additional file 1: Figure S2b, c). This means that while our analysis is unlikely to detect all F2 hybrids among the non-F1 assignments, a F2 hybrid that is detected has a high confidence of being correctly assigned. Confidence in the assignments of F1 and pure individuals was reflected in the estimates of Type I error (false positive rate). In both the *S. perrico* × *S. ghobban* and *S. perrico* × *S. rubroviolaceus* simulations, Type I error rates for F1 were < 0.062; and for the *S. ghobban* × *S. rubroviolaceus* simulations, Type I error rates were < 0.094 (Additional file 5: Table S4). In all simulations, Type I error rates for assignment to the two pure classes were < 0.038. For F2 individuals, the Type I error rate varied substantially among crosses. All the simulated F2 individuals that were called at a critical posterior probability of 0.85 were correctly identified in the *S. perrico* × *S. rubroviolaceus* simulations and the *S. ghobban* × *S. rubroviolaceus* simulations. However, 22% of the simulated F2 from *S. perrico* × *S. ghobban* crosses were assigned incorrectly to the two backcross classes. The Type II error rates (false negative) of the pure and F1 classes were consistent with the high efficiency estimates of these classes (Additional file 5: Table S4), with all being detected at a critical posterior probability of 0.85. Thus, our power simulations reveal that we have high confidence in assignments to the pure and F1 classes, while the nature of deeper generational hybrids remain uncertain.

#### Hybrid zone structure

To contrast the structure of hybrid zones across species pairs and localities, we ran Structure, K = 2 models for each species pair and used the admixture proportions from these models as a hybrid index. Hybrid zones characterized by frequent backcrossing into the parental species are expected to have unimodal distributions of the hybrid index and the breakdown of species into a hybrid swarm, while hybrid zones with limited backcrossing will have bimodal or even trimodal hybrid indices, depending on the frequency of F1 hybrids [42]. Further, if the degree of hybrid breakdown depends on age of divergence between the parental species [43, 44] we predict that the *S. ghobban* × *S. rubroviolaceus* hybrid zone should have a more continuous distribution of hybrid indices, compared to the two other hybrid zones involving older species divergences (*S. perrico* × *S. ghobban* and *S. perrico* × *S. rubroviolaceus*).

**Fig. 4:**
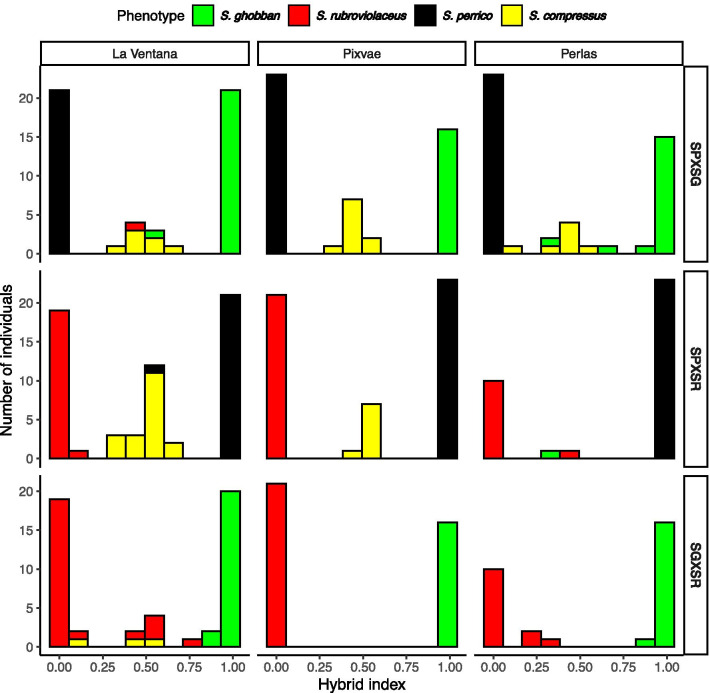
The distribution of hybrid indexes for the three different hybrid zones (rows) and among the three localities (columns). Hybrid indexes are = Q-values from a STRUCTURE, K = 2 model representing each cross. Individuals within each bar are colour coded by phenotype

The structure of the six hybrid zones involving *S. perrico* were either bimodal or trimodal depending on locality, with the distribution of hybrid indices clustering around 0.5 (Fig. 4 top and middle panels). The structure of the three *S. ghobban* × *S. rubroviolaceus* hybrid zones were bimodal, yet these hybrid zones showed the broadest distributions of hybrid indices (Fig. 4 lower panel). These patterns indicate that hybrid zones caused by mating between species with older ages of divergence (*S. perrico* × *S. ghobban* or *S. rubroviolaceus*) were dominated by F1 hybrids, while the hybrid zones caused by mating between the younger species pair: *S. ghobban* × *S. rubroviolaceus*, had a more even mix of F1 hybrids and hybrids of potentially deeper origin.

#### Does rate asymmetry between mitochondrial and nuclear genes explain patterns of mt variation in hybrids?

Age of divergence between species is a primary driver of post-mating isolation via hybrid breakdown but accelerated rates of evolution in mt genomes relative to the nuclear genome can also lead to incompatibilities that are only expressed when females with the “fast” mt DNA mate with males with the “slower” mt DNA [45]. To test for a potential role of “Darwins corollary to Haldane’s rule” in the patterns of mito-nuclear discordance we observed in hybrids we used a comparative approach described in [46].

Estimates of rate asymmetry in mtDNA evolution ($\delta$) vs. nuclear evolution for the three hybrid crosses do not strongly support mitonuclear incompatibility as an explanation for asymmetries in mitochondrial genotypes in hybrids (Table 1). While estimates of ${\delta }_{i}$ were positive in two of the three hybrid pairs (shaded columns in Table 2), the magnitudes were low (< 0.5). Haplogroup B was the most common mt genotype in *S. ghobban* × *S. rubroviolaceus* hybrids and had the slower rate of mt vs. nuclear evolution (${\delta }_{C}=0.109$), a pattern consistent with the mitonuclear DMI model. Yet in both hybrids involving *S. perrico* values of $\delta$ were very close to 0.0. We note that hybrids formed by this species had the strongest mito-nuclear disocordance. In each pair, the *S. perrico* mt haplogroup was either very rare (*S. perrico* × *S. rubroviolaceus*) or not observed at all (*S. perrico* × *S. ghobban*)*.*

**Table 2:** Model selection results from six Migrate models simulating introgression across three species boundaries

Model	M parameters	ln(mL)	LBF	Probability
1	Full model—all	− 4443.47		6.824E−87
2	Sp → Sg Sp ← Sg Sr → Sg Sr ← Sg	− 4394.11	98.72	1.866E−65
3^a^	Sr → Sg	− 4339.68	108.86	8.119E−42
4^a^	Sp → Sg	− 4250.54	178.28	0.004
5	Sp → Sg Sr → Sg	− 4247.52	6.04	0.086
*6*^a^	*Sp *→* Sg* *Sp *←* Sg*	*− 4245.16*	*4.72*	*0.909*

Arrows specify the direction of introgression. Ln(mL) are the log marginal-likelihoods estimated using the Bezier approximation. Log Bayes factors (LBF) are calculated by comparing Model x with the Model x − 1

Sp—*S. perrico*, Sr—*S. rubroviolaceus*, Sg—*S. ghobban*

^a^ Models 3, 4, and 6 include a constant low migration parameter (M = 0.001) to meet the assumptions of connectivity among all populations. Italic text indicates the best fitting model = Model 6

### Q3. Is there evidence for historical gene flow and introgression among species pairs?

#### Migrate models of introgression between three species

To determine historical patterns of introgression among the three biological species we constructed a three population Migrate model [48] by using only “pure” adults. By selecting samples for the model with Structrue Q-values $\ge$ 0.90 we minimized the effect of including any recent backcrosses in the model and their tendency to inflate estimates of introgression. The program Migrate estimates mutation-scaled effective population sizes ($\theta$), and migration and immigration rates (*M*), for each population included in the model. We were primarily interested in differences in *M* among species pairs. For example, are M values higher for older or younger species pairs?

We ran a total of six different Migrate models (Table 2) to assess levels of introgression among the three species and obtained marginal likelihoods and posterior distributions for all parameters. We used the marginal likelihoods of the models and Bayes factors to determine the best fitting model (Model 6), which included only migration between *S. perrico* and *S. ghobban* (Table 2). All other models had lower marginal likelihoods, and the Log Bayes factor between the next best fitting model (Model 5) exceeded 2 which is considered strong evidence for poor model choice [49]. Estimates of gene flow between species in these models could be biased if our filtering threshold for hybrids (Q > 0.10 in > 1 cluster) did not remove individuals that resulted from backcrosses from the samples included in the model populations. To test this possibility, we ran Structure models with K = 3 for all the simulated crosses generated for the NewHybrids power estimation and compared the distribution of Q values for individuals in the five classes. We found that in both the *S. ghobban* × *S. rubroviolaceus* and the *S. perrico* × *S. rubroviolaceus* crosses, backcrosses occasionally had Q values that fell below the filtering threshold (Additional file 1: Figure S3). In the cases of *S. ghobban* × *S. rubroviolaceus* crosses, backcrosses in both directions could have Q < 0.10, and in the case of backcrosses between F1 hybrids and *S. rubroviolaceus* the median value of the distribution was very close to 0.10. while for the *S. perrico* × *S. rubroviolaceus* crosses, a few backcrosses between F1 hybrids and *S. perrico* had Q < 0.10. Thus if bias was significantly impacting the Migrate results, we expect the most bias in gene flow across the species boundary between *S. ghobban* and *S. rubroviolaceus*, yet all models that included *M* parameters between these two species had consistently lower likelihoods (Models 1–3, and 5, Table 2) than models that excluded these parameters (Models 4 and 6). Thus, it does not appear that including backcrossed individuals in the samples used for Migrate models are significantly biasing the results.

An alternative explanation for the allele sharing in the three parental species across both mitochondrial and nuclear loci in *Scarus* is that lineage sorting of ancestral variation is far from complete at the mitochondrial locus and nuclear loci. Coalescent theory predicts that incomplete lineage sorting will be increasingly likely as the splitting time is closer to the present and/or *N*_*e*_ increases. We estimated the ratio: $\frac{t}{{N}_{e}}$ for each of the three biological species based on the three $\theta$ values from the best fitting Migrate model and the splitting times of the clades given in Fig. 1 (Additional file 6: Table S5) and compared these values to the probability calculations of Hudson and Coyne [50]. The ratio of time (in generations) to effective population size = $\frac{t}{{N}_{e}}$ for 0.95 probability of reciprocal monophyly is $\ge$ 2.20 for a mitochondrial locus and $\ge$ 11.8 for five nuclear loci. With the exception of *Scarus ghobban* where $\frac{t}{{N}_{e}}$= 5 at 4.36 Ma, and 7 at 6.60 Ma, the remaining two species have $\frac{t}{{N}_{e}}$ values > and >> 17, strongly decreasing the probability that ancestral genetic variation in *mtCR* or at the four nuclear loci would remain segregating in contemporary populations of *S. rubroviolaceus* and *S. perrico*. For *S. ghobban*, the relatively larger N_e_ value suggests it is plausible that some of the genetic variation we sampled at some nuclear loci is shared across species because it was shared at the time of lineage splitting. Overall, we conclude that the shared nuclear and mt variation among species is most likely explained by hybridization and introgression after contact between these species.

### Q4. What is the relative abundance of the hybrid species *Scarus compressus*, and do the relative abundances of the hybrid varieties vary among localities?

#### Patterns of ecological abundance and relative abundance from field surveys

In visual counts for the three parental species: *S. perrico*, *S. ghobban*, and *S. rubroviolaceus*; we recorded highest abundances at the two Panamanian sites (Pixvae and the Perlas Islands) compared to consistently lower abundance at La Ventana, Baja California (Additional file 7: Table S6). At two localities in the Perlas Islands we observed schools of juvenile *S. ghobban* that exceeded 1000 individuals.

**Fig. 5:**
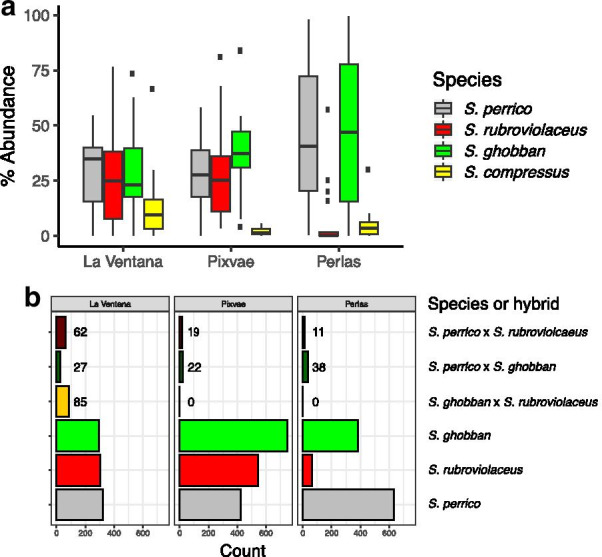
Ecological abundance of three species of *Scarus* parrotfishes and their hybrids among three localities in the Tropical Eastern Pacific. **a** Whisker plots of relative abundance based on field counts of replicate sites within localities and with all life history phases combined. **b** A comparison of the estimated abundance for the three hybrid varieties based on total adult counts (IP and TP phases) for each locality. Numbers to the right of bars for hybrid varieties illustrate the relative differences within and among localities. The abundance of each hybrid class was estimated using the relative frequency of hybrid- and non-hybrid genotypes in the genetic data set

The mean relative abundance of *S. compressus* was < 15% of all *Scarus* individuals at all three sites, but varied significantly among sites (single factor ANOVA: F = 6.88, df = 2, P = 0.002) (Fig. 5a). Tukey’s HSD means comparisons revealed that *S. compressus* had significantly higher relative abundance at La Ventana (14.2% of all *Scarus* individuals) compared to the Perlas Islands (4.7%, P = 0.013), or Pixvae (2.1%, P = 0.003) but that there was no difference in the relative abundance of *S. compressus* between the Perlas Islands and Pixvae (P = 0.718). There were also differences in the relatively abundance of individual parental species among localities, most obviously the rarity of *S. rubroviolaceu*s in the Perlas Islands (Fig. 5a).

#### Patterns of relative abundance of hybrid varieties from the genetic data

The estimated relative abundance of the three hybrids varieties among localities varied considerably (Fig. 5b). For example, we only sampled *S. ghobban* × *S. rubroviolaceus* hybrids from La Ventana, Baja California, and the relative proportion of the two varieties of “*S. compressus*” (*S. perrico* × *S. rubroviolaceus* and *S. perrico* × *S.* *ghobban*) shifted from a high frequency of *S. perrico* × *S. rubroviolaceus* in the north at La Ventana to a high frequency of *S. perrico* × S. *ghobban* in the south, in the Perlas Islands. In some cases, relative hybrid abundance was coupled, and in others de-coupled, to the relative abundances of the three parental species. For example, *Scarus rubroviolaceus* was rarely observed at the northern Perlas Islands and, predictably, the relative estimates of hybrids involving this species there were either very low (*S. perrico* × *S. rubroviolaceus*) or non-existent (S*. ghobban* × *S. rubroviolaceus*). Further, while the three parental species were observed in similar proportions at both La Ventana and Pixvae, *S. ghobban* × *S. rubroviolaceus* hybrids were present only at La Ventana.

#### Observations feeding schools and social behaviour in the field

Size-specific patterns of variation in colouration and sexual expression is common in the Labridae. Initial phase (IP) individuals are smaller, drab in colouration, and typically express female gonads. In contrast, terminal phase (TP) individuals are larger, brightly coloured, and typically express male gonads. We observed multiple individuals of both IP and TP *Scarus compressus* joining large multispecies feeding schools of dozens to scores of adults of varying proportions of the other three species (see *Scarus* hybrid movie, Additional file 8). We also observed schools that included adults of all four species roaming actively across hard reef habitats of La Ventana, Baja California and Pixvae, Panama. Sites in the northern Perlas Islands, Panama lacked *S. rubroviolaceus*, and multispecies schools were limited to other three species in this region of the Perlas Islands. Additional field observations of the colour patterns and social behaviour of each species in the field can be found in Additional file 9.

### Q5. Are male and female *S. compressus* fertile?

To determine if hybrids were reproductively active, we sampled gonad tissues from each species and from initial and terminal colour phases. Our reproductive data show that *S. ghobban*, *S. rubroviolaceus*, *S. compressus*, and, possibly, *S. perrico,* all exhibit size-specific patterns of variation in colouration and sexual expression that are typical of the Labridae. Differences in colouration between intermediate- and large sized *S. perrico* are less pronounced*,* but the largest individuals in a school typically possess a large nuchal hump and are presumably male. Unfortunately, our sample did not include any of these very large individuals.

The fraction of initial phase (IP) and terminal phase (TP) individuals in which sex could be determined (i.e. had recognizable male or female gonads) varied among species, ranging between 48 and 71% (Table 3). Of the sexed individuals, all of the TP *S. compressus* had male gonads (n = 12) and 4 of these individuals (33%) were sexually active. All of the IP *S. compressus* with active gonads were females (n = 10) and 4 (40%) where sexually active. None of the IP *S. compressus* we sampled had male gonads. In both *S. ghobban* and *S. rubroviolaceus* we sampled reproductively active males with either IP or TP colouration. *Scarus rubroviolaceus* showed the highest levels of sexual activity—all the TP males (n = 6) were sexually active and 86% (n = 29) of the IP females were sexually active. There were a small number of reproductively active IP males in *S. ghobban* and *S. rubroviolaceus*, consistent with a diandric mating system.

**Table 3:** Sexual activity between colour phases (IP = initial phase, TP = terminal phase) and species

Species	n	# male		# female		# active males		# active females		% SM
Phase		IP	TP	IP	TP	IP	TP	IP	TP	
*S. compressus*	45	0	12	10	0		4 (33)	4 (40)		49
*S. ghobban*	64	4	2	25	1	2 (50)	1 (50)	14 (56)	0 (0)	50
*S. perrico*	64	5	0	17	0	2 (40)		11 (64)		34
*S. rubroviolaceus*	80	6	4	29	0	6 (100)	1 (25)	25 (86)		49

Data based on visual inspection of dissected fish from La Ventana and Pixvae. Active individuals had either semi or active gonad conditions and % of sample is given in parentheses. See methods for definitions of gonad conditions. % SM = % of the sample that was sexually mature = total number of samples scored for male or female gonads/total number of samples (n) * 100

These data indicate that the hybrid species *S. compressus* is fertile and likely to be actively engaged in reproduction at La Ventana and Pixvae.

### Q6. Is there diagnostic morphological and colour variation among species and hybrid varieties?

Counts of scales and fin rays can be diagnostic of species in some fish groups, but have proven to be less informative in closely related species of parrotfishes, including the *Scarus* complex studied here in the TEP [51]. We compare meristic patterns among the four species from our samples and use colour photographs of post-mortem individuals to retrospectively map colour traits onto hybrid varieties as determined by our Structure, K = 3 admixture model.

#### Meristic variation in morphology

*Scarus ghobban* and *S. rubroviolaceus* had similar distributions of numbers of cheek scales, predorsal scales, and pectoral rays (Additional file 1: Figure S5a–d). On the other hand, *S. perrico* had consistently fewer cheek scales, predorsal scales, and pectoral rays than either *S. ghobban* or *S. rubroviolaceus*. The hybrid, *S. compressus*, had scale and ray counts that either matched one of the parental species, or lay between the two parental species, depending on the meristic trait. These patterns generally matched those observed by Rosenblatt and Hobson in Baja California [51] (Additional file 1: Figure S5a–d). Morphology and meristic traits differentiated the oldest species pairs (e.g. *S. perrico* from either *S. rubroviolaceus* or *S. ghobban*) but could not diagnose the two younger species (*S. rubroviolaceus* from *S. ghobban*). The variation sampled in *S. compressus* was also not informative at diagnosing a unique group.

#### Colour patterns and head shape in hybrids

By mapping genetic identity onto the post-mortem photographs of sampled fish we found three distinct hybrid groups that could be differentiated by colour and colour patterning (Additional file 1: Figure S6a–c). Fishes we initially identified as “*Scarus compressus”* could be divided into two groups from colour and morphological traits observed in photographs. Among *S. perrico* × *S. ghobban* hybrids (Additional file 1: Figure S6a) IP individuals had brownish/green bodies, with lighter flecking on posterior body scales, and less distinct pale blue vertical bars than those of pure IP *S. ghobban*. TP fish had light turquoise bodies, with brown scales at the posterior end, distinctive dark blue margins of the pectoral and anal fins similar to those of pure TP *S. perrico*, plus an orange stripe along the interradial membrane of the dorsal fin equivalent to that seen in pure TP *S. ghobban*. The morphology of the head was also distinctive in TP *S. perrico* × *S. ghobban* hybrids, with a clear notch in front of the eyes, separating the snout and upper head, similar to that seen in pure large individuals of *S. perrico*. In comparison, TP *S. ghobban* had a symmetrical and triangular shaped head, without any forehead notch. A second phenotypic group consisted of *S. perrico* × *S. rubroviolaceus* hybrids (Additional file 1: Figure S6b). IP fish of this type had red-brown bodies with light flecks on body scales in the rear half of the body similar to IP *S. rubroviolaceus*, plus distinct blue margins along the dorsal and anal fins that are seen in pure IP *S. ghobban* but not in pure IP *S. rubroviolaceus*. TP *S. perrico* × *S. rubroviolaceus* hybrids had bright blue heads, and light turquoise bodies with fewer brown scales on the body than TP *S. perrico* × *S. ghobban*. The head shape of TP *S. perrico* × *S. rubroviolaceus* hybrids was bullet shaped like that of pure TP *S. rubroviolaceus* and lacked the distinctive forehead notch found in large *S. perrico*.

Hybrids of *S. rubroviolaceus* × *S. ghobban* also formed a distinct phenotypic group in each colour phase (Additional file 1: Figure S6c). IP fish were very similar to those of *S. rubroviolaceus*, with uniform red-brown bodies, but also had blue margins on dorsal and anal fins similar to IP *S. ghobban* and unlike IP *S. rubroviolaceus*. IP fish also had blue colouration on the sides of the beak. TP fish were distinctive, with bright blue body scales, a lunate caudal fin, and a symmetric, triangular head.

These differences in colour and morphology were consistent among the sites, and once recognized, provided useful information for discriminating the three hybrid classes from the parental species in the field.

## Discussion

### The nature of Scarus compressus

Our genetic data strongly suggest that *Scarus compressus* (Osburn and Nichols, 1916) is not a biological species (sensu [52, 53]), but is the product of a complex of hybrid relationships involving mating between the Tropical Eastern Pacific endemic *S. perrico* and two other, distantly related congeners: *S. ghobban* and *S. rubroviolaceus*. Our large sample of *S. compressus* individuals had a high probability of heterozygosity across all four nuclear loci, and the mitochondrial and nuclear alleles sampled in *S. compressus* were common in the three putative parental species (e.g. Fig. 2a–e). A Structure model identified three clusters associated with the parental species: *S. ghobban*, *S. rubroviolaceus*, and *S. perrico*; while all the *S. compressus* samples had admixed assignments (Fig. 2f). Admixed individuals had morphological traits and colour patterning that reflected contributions from the two parental species identified by the three Structure clusters, and we recognized three groups of distinct hybrids in our sample based on differences in morphology and colour. Our NewHybrids models reliably assigned nearly all the *S. compressus* samples to the F1 hybrid class, indicating that breakdowns in premating isolation are continuously generating new hybrids in this system. A general lack of > F1 hybrids indicate that post-mating factors are preventing the dilution of the parental species into a hybrid swarm. Nonetheless, our Migrate analysis indicates that over deep evolutionary time scales (100 s of thousands of years), some introgression has occurred between one of the oldest species pairs: *S. perrico* and *S. ghobban*. The ages of the divergence events for each species pair (Fig. 1) combined with the effective population sizes for each species (Additional file 6: Table S5) suggest that little of the shared genetic variation between species can be explained by the process of incomplete lineage sorting. Thus, our data strongly support a hypothesis of contemporary hybridization between *S. perrico* × *S. ghobban* and *S. perrico* × S *rubroviolaceus* as an explanation for the phenotypes described as the species *S. compressus* in the TEP.

While our data collectively strongly support a “three-species hybrid complex”, an alternative explanation involving high rates of hybridization among four species is also possible. In this “four-species” scenario, a small fraction of the *S. compressus* population at each locality is a recently derived species from one of the other species in the complex, and this fourth species hybridizes freely within the complex. If this fourth species was derived within the last tens of thousands of years, and mated non-discriminately within the complex, it could produce the patterns and levels of heterozygosity observed at our four nuclear loci in *S. compressus* and may remain undetected in the admixture analysis. A central challenge to the “four-species hybrid complex” hypothesis is that the probability of intraspecific mating would need to be lower than the probability of interspecific mating in the fourth species in order to be consistent with the extremely high levels of multilocus heterozygosity we observed in our *S. compressus* sample (see Additional file 10 for quantitative estimates). Low intra- vs. inter-specific mating raises the question of how species cohesiveness could be maintained, particularly given the ecological rarity of *S. compressus* at many sites (Fig. 5a). While a four-species hybrid complex appears to be less parsimonious with our genetic and ecological data than a three-species hybrid complex, more extensive genomic sampling is required to strongly reject one of these alternatives.

#### The breakdown of pre-mating isolation

Differences in the structure and function of TEP rocky reefs compared to coral reefs may be strongly limiting ecological opportunity while simultaneously increasing opportunities for hybridization. Ecological niche space for parrotfishes in the TEP is constrained by a general lack of active coral reef construction. Rather, the vast majority of primary grazing surfaces are associated with rocky benches and boulders, and the TEP largely lacks the structural and erosional niche dimensions associated with coral reefs that is positively related to species- and functional diversity [54, 55]. Powerful excavating species like *S. perrico* and *S. rubroviolaceus* may thrive in the rocky-reef habitats of the TEP because of jaw morphologies and musculature that can effectively remove low-lying encrusting epibionts from smooth rock surfaces. In contrast, *Scarus ghobban* forages over soft sediments as well as on hard substrata and does not appear to be ecologically limited by the lack of coral reef substrata as coral-reef dependent *Scarus* species. These dramatic differences in structure and function of benthic habitats compared to those on coral reefs are likely to act as an ecological filter by constraining the functional dimensions available to parrotfishes in the TEP. In contrast, the coral-rich habitats of Greater Caribbean on the opposite side of the Isthmus of Panama to the TEP support six species of endemic *Scarus* and five species of endemics of the Atlantic parrotfish clade, *Sparisoma*. Yet the TEP has only one endemic *Scarus* species (*S. perrico*) and no species of *Sparisoma*. The only other genus with an Atlantic-clade species on each side of the isthmus is *Nicholsina*, which is ecologically distinct from *Scarus*. Our limited niche space hypothesis posits that a paucity of ecological opportunities for parrotfishes in the TEP can account for greatly reduced diversity of Atlantic parrotfish clades in that region, and that both new colonists arriving from more developed coral-reef systems in the central Pacific, and phenotypes generated by hybridization in the TEP, must pass through this ecological filter to be successful there.

Little is yet known about the mating systems of our three study species. Mating aggregations have been observed for both *S. rubroviolaceus* and *S. ghobban* in the West Pacific [56]. The only observations of spawning within this species complex in the TEP that we are aware of are those of Rosenblatt and Hobson [51], who described the three species aggregating at the same time in the same temporary spawning area in the Gulf of California. They also observed an aborted heterospecific pair-spawning attempt between *S. rubroviolaceus* and *S. ghobban*, which involved a TP *S. rubroviolaceus* momentarily pairing with an IP *S. ghobban*. In our experience, heterospecific harems like those involving IP *S. compressus* acting as members of harems of *S. rubroviolaceus* that we repeatedly observed in Baja California, are generally rare in the parrotfishes. Rosenblatt and Hobson’s observation illustrates the point that mate control is most effective during pair-spawning behaviour, and the mating systems of territorial haremic species like *S. rubroviolaceus* may tend to reduce mating mistakes by male and female behaviour. In contrast, lack of formation of harem groups, which is seen in *S. perrico* and *S. ghobban* facilitates participation of multiple males in spawning events, as well as non-territorial IP males interfering in the spawning of TP territorial males, and the occurrence of heterospecific mistakes.

**Fig. 6:**
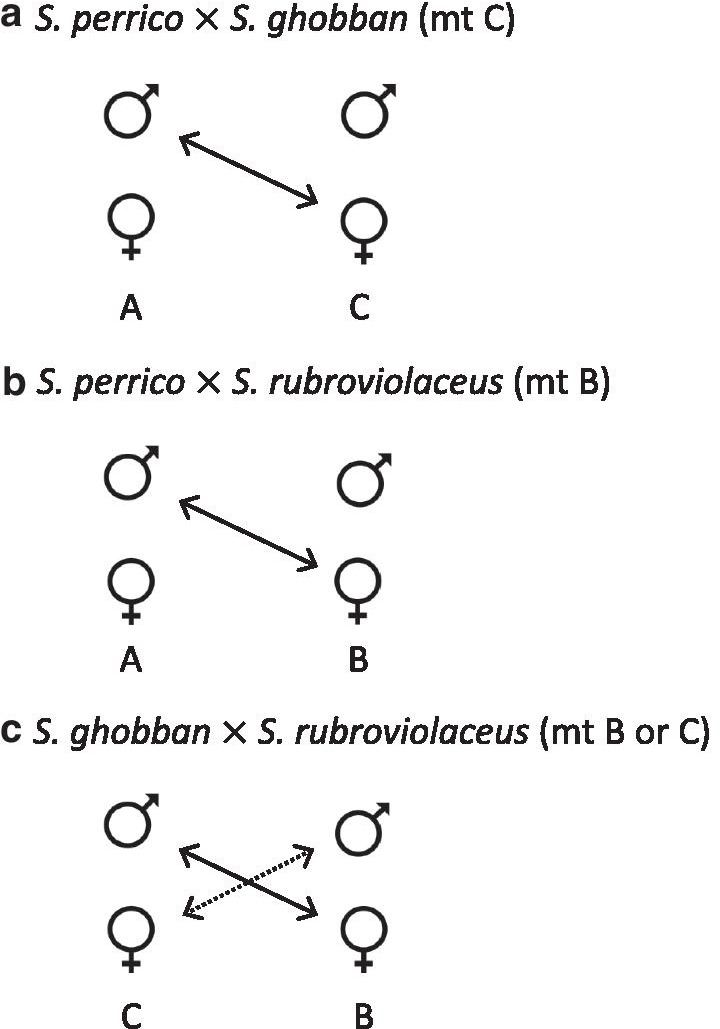
Asymmetry in reciprocal hybrid crosses inferred from mtDNA genotypes, assuming uniparental inheritance through females. The dominant mitochondrial haplogroup (as in Fig. 2) observed in each hybrid class are indicated in parentheses following the cross name. Species-specific mtDNA haplogroups are indicated below the species columns for each cross. Solid lines on arrows indicate the most common cross, dotted lines indicate less common cross

Patterns of mtDNA asymmetry in all three hybrid pairs observed in the TEP, combined with alternative male spawning tactics seen in mating systems of parrotfish with different social systems, suggest an important role for indiscriminate spawning behaviour by male *S. perrico,* especially, and male *S. ghobban*. In both of the hybrid crosses involving *S. perrico*, mtDNA genotypes indicate that females of either *S. ghobban* or *S. rubroviolaceus* are mating with male *S. perrico* (Fig. 6). This asymmetric mating inference assumes that mtDNA asymmetries are set by mating, and not by Dobshansky–Muller incompatibilities (DMIs) that lead to differential viability of reciprocal crosses (e.g. [57]). We found no evidence of accelerated mtDNA evolution in *S. perrico* compared to *S. ghobban* and *S. rubroviolaceus*, an indirect test of a mechanism involving DMIs that leads to differential viability of reciprocal crosses [45]. We hypothesize that indiscriminate mating by male *S. perrico* could be involved in two ways. First, *S. perrico* males could be interfering in the pair spawning events of these latter two species. Second, during group- spawning activity, male *S. perrico* may actively seek to induce spawning in reproductively active females of the other two species. Male–male competition during group spawning can be intense, and highly coercive males often jostle for optimal position around single sexually active females [58, 59], and we have observed cross-species courtship by male labrids in other contexts. The strong bias of male *S. perrico* dominating interspecific mating also raises the questions of why it occurs in the environmental context of the TEP? We suggest that a long period of isolation of *S. perrico* as the only *Scarus* species in the TEP may have relaxed selection for strong mate discrimination tendencies seen in more speciose assemblages elsewhere.

#### The biogeography of the Scarus hybrid zone

All evidence suggests that the contact zone in the TEP that produced the region-wide hybrid complex considered here developed after an eastward expansion of two Indo- and Central Pacific species (*S. ghobban* and *S. rubroviolaceus*) that colonized the entire geographic range of the TEP endemic *S. perrico*. The structure of this hybrid complex is unique among reef fishes in general, where the majority of interspecific hybridization has been documented at biogeographic suture zones (sensu [60]), regions where groups of formally allopatric populations or sister species come into secondary contact (reviewed in [25]). In the TEP *Scarus* complex, the biogeographic ranges of all three parental species completely overlap and all three species often co-occur in mixed feeding schools. None of the parental species pairs are recently derived or are sister species. In fact the youngest species pair: *S. ghobban* and *S. rubroviolaceus* are estimated to have shared a most recent common ancestor over 4 Ma. These two species have nearly identical range distributions, both occurring across the Indian and Pacific Oceans [35]. Parallel hybrid phenomena may occur in the angelfishes (Pomacanthidae). A recent survey of hybridization within the Pomacanthidae found that 59% of 37 hybridizing species pairs had sympatric distributions (defined by $\ge$ 50% range overlap) and some species pairs had older divergence dates as indicated by *mtCO1* divergence between 5 and 8% [24]. Intriguingly, protogynous hermaphroditism is common in both the parrotfishes and the angelfishes, with mating system attributes that may facilitate heterospecific mating, as described under *The breakdown of pre-mating isolation.* On the other hand, in 85% (n = 20) of angelfish hybrid zones where observations of abundance were available, one or both parental species were rare [24], an ecological pattern that clearly does not apply to the *Scarus* hybrid complex in the TEP where all three parental species are conspicuously abundant (Fig. 5).

We do not know the width of the westward “tail” of the cline since we have yet to sample the genomes of *S. ghobban* and *S. rubroviolaceus* from other Indo-Pacific localities. While F1 hybrid phenotypes (e.g. *S. compressus*) are undocumented beyond the TEP, the dispersal capacity of *S. ghobban* and *S. rubroviolaceus*, as exemplified by their ability to spread from Africa to the Americas and cross the enormous expanse of the EPB and establish populations in the TEP, provides opportunities for migration outside the TEP and the formation of an exceptionally wide genetic cline [37]. Within the TEP, we also do not know if hybrids are dispersing broadly throughout the TEP, or if there is substructure among smaller regions. Lessios and Baums [61] found high gene flow across the latitudinal range of the continental shore of the TEP in *S. ghobban* and *S. rubroviolaceus*, but evidence for genetic structure between offshore islands and the mainland. The width of hybrid zones in other tropical reef-fish systems may expand over similar spatial scales and appear to be driven by dispersal. Wilcox et al. [23] have found evidence for a hybrid lionfish species (genus *Pterois*) whose range extends from western Indonesia to southern Japan, a similar geographic scale as that described here. The large geographic scales of these hybrid zones differ markedly from the scale of most other marine and terrestrial hybrid zones in which genetic clines are thought to be maintained by a balance of dispersal away from the spatially limited cline centre, and natural selection against hybrids [62]. In contrast, massive scale of the TEP *Scarus* and Western Pacific *Pterois* hybrid zones appear to largely reflect effects of high dispersal capabilities and, perhaps, weaker selection against hybrids, because parental species and hybrid genotypes co-occur throughout a single zone stretching across 1000 s of kilometres.

Hybridization between *S. ghobban* × *S. rubroviolaceus* would be intriguing to explore outside the TEP since these both species occur all the way to coastal Africa, often in sympatry. Does the unique evolutionary history of the TEP create an ecological hotbed for the breakdown of pre-mating isolation in parrotfishes? Or does hybridization occur more generally in these and other parrotfish species as well, at low enough levels to have largely passed unnoticed? With regards to the latter question, there is only one other documented case of parrotfish hybrids that we are aware of, between *Chlorurus sordidus* × *C. perspiculatus* in Hawaii, inferred from phenotypic observations from a handful of TP phenotypes with obvious mixtures of the TP colouration of the putative parent species [63]. While morphological variation indicates that the frequency of hybrids in this case may not be high, that impression comes from comparison of TP coloration, and interspecific differences are much more obvious in TP than IP colouration. It is further intriguing that these two parent species are also not closely related, estimated to share a common ancestor ~ 5.82 Ma [34], and that it is occurring in another peripheral region with a relatively small *Scarus* fauna.

## Conclusions

We have documented a hybrid complex of parrotfishes within the Tropical Eastern Pacific that is most likely the result of ongoing hybridization between three divergent species. We found differences in the frequency of hybrid classes consistent with age of divergence: the fewest deeper generation hybrids (> F1) resulted from mating between species pairs whose ancestors diverged > 6 Ma. In contrast, mating between a species pair that diverged 4.36 Ma, had a more even distribution of F1 hybrids and deeper generation hybrids. We suggest that the breakdown of pre-zygotic isolation between the endemic *S. perrico* and the other two species, and the lack of *S. perrico* mt genotypes in hybrids is the result of historically weak natural selection for accurate species mate discrimination by male *S. perrico*, the product of a long period of isolation as the sole member of its genus in the TEP. Male mating behaviours that involve interference by males in others spawning events are probably important in generating heterospecific “mistakes” in this system. However, we recognize that as yet we know little about the mating systems of these three species. Given the diversity of social systems and flexibility of spawning strategies exhibited by male parrotfishes [64], this system of hybridizing *Scarus* species in the TEP presents an excellent model to understand the evolutionary and ecological factors the contribute to pre-mating breakdown among reef fishes, and the evolutionary consequences of such breakdown. Despite the consistent levels of hybridization in this system, species boundaries remain intact, suggesting that both pre-mating (e.g. mating behaviour) and post-mating processes such reduced fitness in F2 offspring due to the accumulation of DMIs, contribute to maintaining species diversity in this complex.

## Methods

### Q1. Does contemporary hybridization explain *Scarus compressus*?

#### Sampling

We collected samples for genetic analyses from three localities that broadly span the TEP (Additional file 1: Figure S1): (i) the Perlas Islands, in the Bay of Panama; (ii) Pixvae, on the southeastern corner of the Gulf of Chriqui, Panama, almost 300 km from the Perlas Islands; and (iii) La Ventana, Baja California Sur, on the southeast coast of the Baja Peninsula, 3200 km from the Panama localities. These sites are representative of the dominant nearshore habitat found in the coastal TEP, consisting mostly of rocky reefs with small patches of encrusting corals, and a few small, scattered coral reefs. Significant coral accretion is rare in the TEP, which has only ~ 25 km^2^ of structural coral reef, and rocky cliffs, platforms, ledges, boulders and cobbles provide most of the structural habitat for nearshore fishes at these sites. At each of the three localities, we made collections of individual fishes for downstream morphological, reproductive, and genetic analyses. We used spearguns and pole-spears to sample a broad range of sizes and sexual phases and kept all fish on ice until processing on the shore. On shore, each fish was photographed, measured for standard length, and either a liver, or a fin clip tissue sample was collected for genetic analyses. Tissue samples were fixed in 95% EtOH for 24 h, and replaced with new 95% EtOH for long term storage. In addition, otoliths and skeletal material were collected from La Ventana samples and archived at the Autonomous University of Baja California Sur.

#### Sequencing, phasing, and admixture analysis

We Sanger sequenced portions of four nuclear genes—*rag2*, *Tmo4c4*, *bmp4*, and *Dlx2* [39] and the mitochondrial control region *mtCR* [65]. The primers, annealing temperatures, and the gene features of our markers are listed in Additional file 11: Table S7. DNA was extracted from tissue samples using Qiagen DNeasy tissue extraction kits (Qiagen, Valencia, CA), and sequenced by the GENEWIZ sequencing laboratory, South Plainfield, NJ. To identify haplotypes from the Sanger reads we used the software PHASE 2.1.1 [66] to identify the most likely haplotypes, and used the two haplotypes with highest credibility for downstream genotyping and Migrate models. We phased 78.5% of the heterozygous nuclear sequences (n = 400) with a credibility of 1.0 and 95.8% of the nuclear data with a credibility $\ge$ 0.95. Complete phased alignments for each locus with credibility scores are available from Dryad. To call genotypes at each locus, we constructed TCS networks [67] with PopART [68] from each phased alignment and generated lists of identical sequences for each node (equivalent to a unique haplotype). For each locus, we used sequential integers as allele names, and the genotyped data set is available on Dryad. For the nuclear data set we successfully genotyped 97.3% of the 4 locus × 244 sample panel, and for the *mtCR* gene we haplotyped 97.1% of 244 samples. Summary statistics for calculated for all the phased sequences and the genotyped data. We estimated the number of segregating sites (*S*), theta ($\theta$) and nucleotide diversity ($\pi$) for each gene sampled from four species at three localities using the R package Pegas [69]; and tested for linkage disequilibrium (LD) between pairs of nuclear loci for each species sampled from three localities with Genepop v4.7 [70]. To control for false discovery in large tables, we used the q-value approach [71] as implemented in the R Package qvalue [72].

We used the admixture model of Structure [38] to determine the best number of populations that fit our nuclear data (*K*), and to determine admixture proportions among individuals (*Q*), indicating hybrids. If *Scarus compressus* is the result of ongoing hybridisation among the other three species, we expect K = 3, with each population representing one of the biological species. The *S. compressus* samples are expected be admixtures of the other three populations depending on the identity of the parents. Alternatively, if *S. compressus* is a biological species, we expect K = 4 with each population representing one of the biological species, and any hybridization events represented by admixed individuals. We ran 10 replicate simulations for K values ranging from 1 to 6. Each replicate simulation started with 10^4^ burnin steps followed by 5 × 10^5^ sampling steps, and we used the uncorrelated allele frequency model and no prior information on phenotypes (e.g. species identification) for each simulation. To assess the best fitting model to the data, we used the Evanno method [73] as implemented in the software Structure Harvester [74]. After identifying the best fitting model (K = 3, see “Results”) we ran an additional 20 simulations for K = 3, then averaged the results of the output files using CLUMPAK [75].

### Q2. What is the structure of the three hybrid zones with respect to F1 and > F1 hybrids, and does hybrid zone structure depend on the age of divergence between the parental species?

#### Hybrid assignment—NewHybrids

While the admixture proportions for individual samples (*Q* values) from the Structure model provide estimates of the genomic contributions of different species, they cannot resolve the depth of hybridization and therefore the class of hybrid event. For example, an F1 and F2 hybrid will have the same genomic contributions from the two parental species. We therefore used the nuclear data and an explicit model of hybridization that can identify four hybrid classes, implemented in the program NewHybrids [40]. This Bayesian assignment model uses the prior information from parental genotypes to calculate the posterior probability that a putative hybrid genotype belongs to one of four hybrid classes: first generation hybrid (F1), second generation hybrid (F1 × F1 = F2), and one of two backcrosses (F1 × P1 or P2, where P1 and P2 are the parental species). Since NewHybrids can only consider two species at a time, we ran three models, with each representing a specific cross and the putative hybrids. To select the individuals for each specific model representing one of three crosses (1. *S. perrico* × *S. ghobban*, 2. *S. perrico* × *S. rubroviolaceus,* 3. *S. ghobban* × *S. rubroviolaceus*) we used the K = 3 Structure model (described in the previous section) to sort individual samples into pure individuals if the Q value of one of the three clusters $\ge$ 0.90, and hybrid individuals if Q value from one of the three clusters < 0.90. Hybrid individuals were further sorted into one of the three crosses by evaluating the distribution of Q across the three clusters. The three Q-values were ranked, and the two species clusters with the highest Q-values were used to assign that hybrid to a specific cross. For each model we ran 10^4^ burnin steps followed by 5 × 10^5^ sampling steps using the parallel version of NewHybrids [76] and the Bowdoin College High Performance Cluster. Power to detect and correctly assign hybrid classes depends on the number of unlinked loci in the marker panel and the differences in allele frequencies between the two parental populations at each locus [40]. To determine the power of our nuclear data set to detect the four hybrid classes in each cross we ran simulations using the Hybriddetective workflow [41] in the R environment (see Additional file 12).

#### Structure of hybrid zones using a hybrid index

To visualize the structure of the three hybrid zones across the three sites [42] we calculated a hybrid index for each cross by constructing three, two-species Structure models with the same individuals (parentals and hybrids) used in each NewHybrids model. We ran 20 replicate simulations for K = 2, and used CLUMPAK to average Q values across runs. We then plotted these Q values as frequency distributions for all individuals collected from each of the three localities.

#### Rate asymmetry between mitochondrial and nuclear genes

To test for a potential role of “Darwins corollary” as a mechanism impacting post-mating survivorship of specific male–female crosses (a class of DMIs) we used a comparative approach described in [46, 46]. See Additional file 13 for the details of this analysis.

### Q3. Is there evidence for historical gene flow and introgression among species pairs?

To determine historical patterns of introgression among the three species we constructed a three population Migrate model [48] by using only “pure” adults (Q $\ge$ 0.90) from the three species and three localities. The program Migrate estimates mutation-scaled effective population sizes ($\theta$), and migration and immigration rates (*M*), for each population included in the model and we were interested in differences in *M* among species pairs. For example, are M values higher for older or younger species pairs? We chose not to include splitting time parameters representing species divergence in these models because most of the ratios of splitting times to effective population size were very large (see “Discussion”) and little genetic signal of these old splitting events will remain in the gene genealogies. To evaluate the best fitting models in terms of number of migration parameters included in the model, we ran the full model with all possible *M* parameters (3 $\theta$ parameters and 8 M parameters), and five other models which included fewer migration parameters (see Table 2). We chose the best model by a Bayes Factor approach, which uses the log- probability of the data given the model (marginal likelihood) [77]. To run each model, we conducted a few short preliminary runs of the full model to obtain estimates of *M* and to verify that parameter estimates were converging. After obtaining estimates of the 8 M parameters, we ran all models again using these *M* estimates as starting points, and 5 × 10^5^ burnin steps followed by 5 × 10^6^ sampling steps. Runs were actually longer than this since genealogies were sampled every 100 steps to minimize autocorrelation. We replicated each chain ten times and used four heated chains per replicate to improve the sampling of parameter space. We checked for adequate sampling by visualizing the posterior distributions at the ends of runs for all parameters and confirming unimodal distributions. All model runs where conducted on the Bowdoin High Performance Cluster with the parallel version of Migrate (v. 3.7.2) [78]. A source of bias on $\theta$ and *M* in our results could result from incorrectly including back-cross individuals in the samples of “pure” parental individuals if the true distributions of Q for backcrosses exceeded the parental threshold of 0.90. To determine the presence and magnitude of such an effect ran a Structure, K = 3 model using the simulated genotypes (pure, F1, F2, and backcrosses) from the NewHybrids power analysis, and compared the distribution of Q-values for each class of cross. We used the averaged Q values from 10 replicate simulations of this model to calculate the fraction of backcross individuals for each cross that had Q values > 0.90. Any wrongly classified individuals would positively bias migration rates in the direction of the backcross, for example if a backcross individual that resulted from a mating between an F1 hybrid and a pure individual of *Scarus perrico* were included in the sample of pure *S. perrico* gene copies for Migrate models, we expect that the M parameter representing gene flow into *S. perrico* to be upwardly biased. In this way we used simulation results to interpret parameter estimates and model selection results.

### Q4. What is the relative abundance of the hybrid species *Scarus compressus*, and do the relative abundances of the hybrid varieties vary among localities?

To compare the relative abundance of species among the three localities, replicate censuses were conducted at 16–20 sites within each locality. To ensure consistency of classification of phenotypes all censuses were done by DRR. At each site, DRR made counts of all four species and noted three sexual phases (juvenile, initial phase or IP, and terminal phase or TP) while diving on snorkel and censused all reef habitat between 1 and 15 m water depth. Surveys typically lasted 1–1.5 h and were conducted via a unidirectional snorkel parallel to the shore. In the Perlas Islands census locations were scattered across various islands along the western side of the 50 km length of the island chain. At Pixvae censuses were made along a ~ 22 km stretch of coastline and adjacent islands. At La Ventana censuses were made along 27 km of the Peninsula shore, plus locations scattered around Isla Cerralvo, a 30 km-long island ~ 10 km offshore from the Peninsula. We compared the abundance of different species using both individual counts and relative measures. For counts, we considered sites as replicate measures of locality abundance and the data was expressed as the # of individuals site^−1^. However, since the area of habitat covered varied during each census and was not quantified, our focus is primarily on the relative abundance of each species. At the Perlas Islands, we counted 31–1766 fish per site (n = 22 sites), at Pixvae we counted 59–356 fish per site (n = 16 sites), and at La Ventana we counted 9–189 fish per site (n = 17 sites). For proportional data we used ANOVA models to determine if species composition changed among sites.

### Q5. Are male and female *S. compressus* fertile?

We determined the reproductive condition of fishes collected at La Ventana and Pixvae by dissection of the gonads. Sexually mature fish were distinguished by the presence of mature gonads and sex was distinguished by gonad colour and texture: ovaries were translucent whitish to pinkish and granular in appearance, while testes were bright white and of uniform consistency in appearance. For sexually mature fish, three sexual activity states were scored: (i) inactive—sex is recognizable, but gonads are very small, no obvious eggs, no sperm expressible; (ii) semi active—ovaries enlarged, but relatively small, without obvious eggs, testes enlarged, but no free sperm; (iii) active—large gonads, ovaries with obvious eggs, testes from which sperm could be expressed with pressure on the belly. We used these states to determine if hybrid phenotypes were as fertile as the three parental species, and to compare levels of concurrent sexual activity among species. No running ripe females of any species were collected at any site, and there are no published accounts of spawning by any of these species in the TEP.

### Q6. Is there diagnostic morphological or colour variation among species and hybrid varieties?

We collected data on meristic traits from samples of all four species from the Perlas Islands. We counted the number of scales in each of three cheek scale rows, the number of median predorsal scales, and the number of unbranched and branched rays in the pectoral fins. We graphically compared frequency distributions of our scale and fin counts to those published by [51] from samples collected in the Cocos Islands, Gulf of California, and the Galapagos Islands. To compare colour variation between hybrids we used the photographs of hybrid samples as indicated by admixture proportions from the K = 3 Structure model (see “Results”—Q1), and grouped photographs into the three hybrid crosses: 1. *S. perrico* × *S. ghobban*, 2. *S. perrico* × *S. rubroviolaceus,* 3. *S. ghobban* × *S. rubroviolaceus.* We visually identified consistent colour or morphological features within crosses, that were also unique among crosses.

## Supplementary Information

**Additional file 1.** Contains additional figures S1–6 referenced in the text. **Figure S1.** Map of the coastal Tropical Eastern Pacific and three sampling localities. **Figure S2a–c.** Power results from simulations. **Figure S3.** Structure simulations, Q values. **Figure S4.** Phylogenetic hypothesis based on nuclear and mitochondrial genes. **Figure S5a–d.** Frequency distributions of meristic traits for four species in the TEP *Scarus* complex. **Figure S6a–c.** Post-mortem photographs and admixture proportions of pure individuals and hybrids.

**Additional file 2: Table S1.** Molecular diversity among species and localities.

**Additional file 3: Table S2.** Tests of linkage disequilibrium (LD) among sites for each species.

**Additional file 4: Table S3.** Results from Evanno method to choose the best Structure model.

**Additional file 5: Table S4.** Type I and II error in simulations.

**Additional file 6: Table S5.** Coalescence estimates based on Migrate Model 6 $\theta$ estimates.

**Additional file 7: Table S6.** Count data for species, sites, and localities.

**Additional file 8.** *Scarus* hybrid movie.

**Additional file 9.** Observations of colour patterns and social behaviour in the field.

**Additional file 10. **Expected heterozygosity with mixed intra- and interspecific mating.

**Additional file 11: Table S7.** PCR primers and conditions.

**Additional file 12.** The power of hybrid assignment.

**Additional file 13.** Testing Darwin’s corollary.

## Data Availability

Mitochondrial and nuclear sequence data is available from GenBank under PopSet #s: 1635813356 (*Tmo4c4*), 1635812509 (*bmp4*), 1635812955 (*rag2*), 1635812058 (*Dlx2*), and 1480619283 (*mtCR*). The datasets supporting the conclusions of this article are available in the Dryad repository, https://doi.org/10.5061/dryad.jwstqjq69.

## Abbreviations

DMIs: Dobshansky–Muller incompatibilities
Ma: Million years ago
mt DNA: Mitochondrial DNA
TEP: Tropical Eastern Pacific
EPB: Eastern Pacific Barrier
