# Supplementary material for: The origin of the parrotfish species Scarus compressus in the Tropical Eastern Pacific: region-wide hybridization between ancient species pairs
Source: BMC Ecol Evol. 2021 Jan 21;21:7. doi: 10.1186/s12862-020-01731-3 (PMC7853319; doi:10.1186/s12862-020-01731-3)
Supplement: Supplementary file 1 — Additional file 1. Contains additional figures S1–6 referenced in the text. Figure S1. Map of the coastal Tropical Eastern Pacific and three sampling localities. Figure S2a–c. Power results from simulations. Figure S3. Structure simulations, Q values. Figure S4. Phylogenetic hypothesis based on nuclear and mitochondrial genes. Figure S5a–d. Frequency distributions of meristic traits for four species in the TEP Scarus complex. Figure S6a–c. Post-mortem photographs and admixture proportions of pure individuals and hybrids. [file 12862_2020_1731_MOESM1_ESM.docx]

**Supplemental Material**

Figures S1 – S6, Additional file 1

Figure S1. Map of the geographic extent of the Tropical Eastern Pacific and location of three sampling localities. Offshore islands and island groups are not illustrated, and include Clipperton Atoll, the Revillagigido Islands, Cocos Island, and the Galapagos Islands.

Figure S2a. Power of the simulated data to detect and accurately call individuals that belong to two parental classes and four hybrid classes (rows) in three different hybrid crosses (columns). Species abbreviations in crosses are as follows: SP = *S. perrico*, SR= *S. ghobban*, SR= *S. rubroviolaceus.*

Figure S2b. Efficiency of the simulated data to detect individuals in two parental classes and four hybrid classes (rows) in three different hybrid crosses (columns). Species abbreviations in crosses are as follows: SP = *S. perrico*, SR= *S. ghobban*, SR= *S. rubroviolaceus.*

Figure S2c. Accuracy of the simulated data to correctly assign individuals to two parental classes and four hybrid classes in three different hybrid crosses. Species abbreviations in crosses are as follows: SP = *S. perrico*, SR= *S. ghobban*, SR= *S. rubroviolaceus.*

Figure S3. STRUCTURE Q-values for four locus genotypes that result from two pure and four hybrid classes by three simulated crosses. Cross IDs are indicated on the right margin of each panel. Species abbreviations in crosses are as follows: SP = *S. perrico*, SR= *S. ghobban*, SR= *S. rubroviolaceus.* Note simulated backcrosses in the *S. ghobban* **×** *S. rubroviolaceus* cross often have Q values > 0.90.

**

Figure S4. Phylogenetic hypothesis used to estimate relative rates of mt vs. nuclear evolution. Species at tips indicated in red are focal taxa. The labelled internal nodes were used to estimate gene-specific branch lengths for the two *S. perrico* species pairs (node A) and the *S. ghobban* × *S. rubrovioalceus* species pair (node B). Labels to the right of nodes indicate % bootstrap support from 600 ML trees.


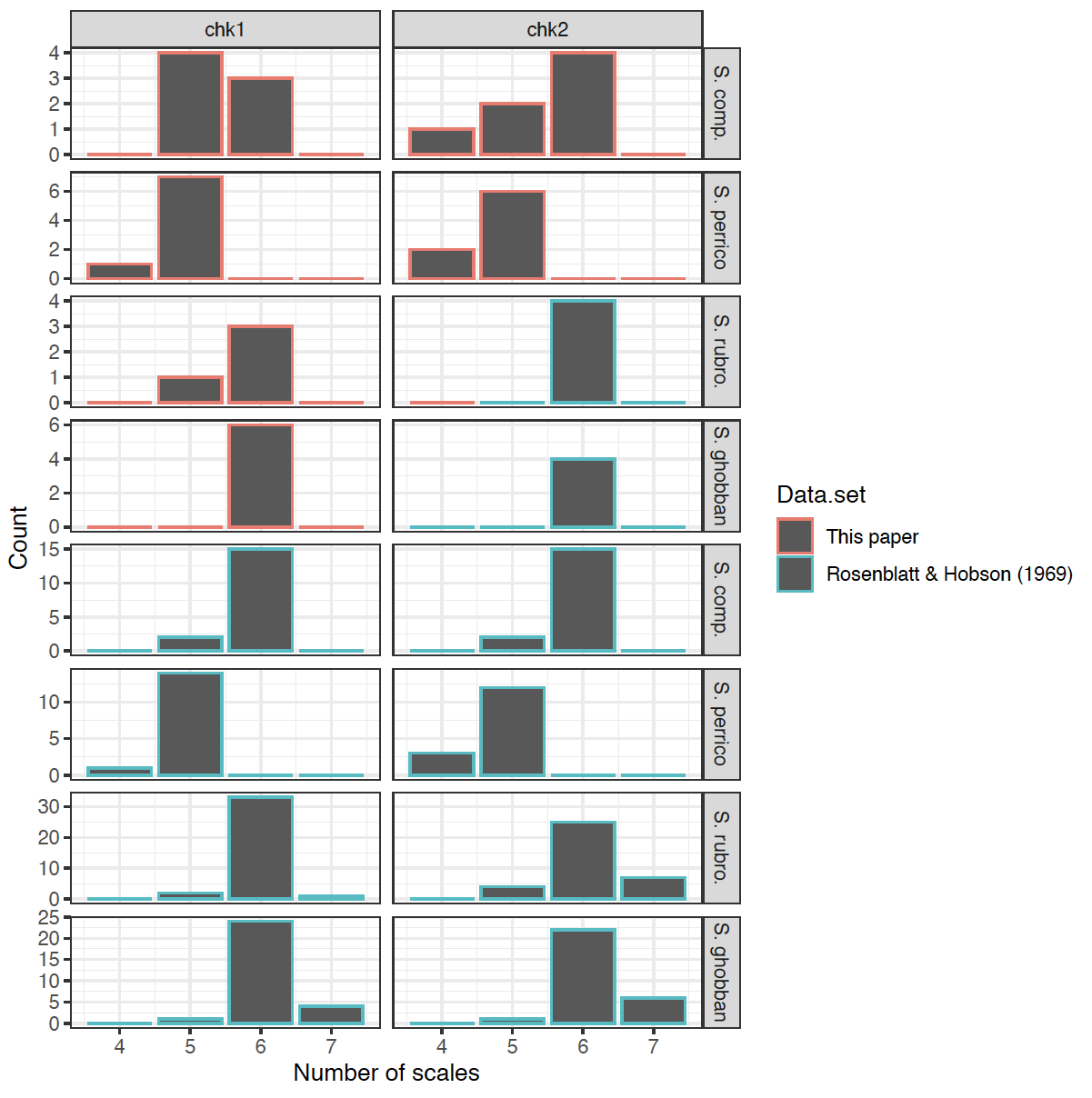


Figure S5a. Meristic data. Frequency distributions for scale counts in cheek scale rows 1 (chk1) and 2 (chk 2). Upper panels with bars with red outline are data from samples collected in the Perlas Islands. Lower panels with bars and blue outlines are data from Rosenblatt, R. H., and E. S. Hobson. 1969. Parrotfishes (Scaridae) of the eastern Pacific, with a generic rearrangement of the Scarinae. Copeia 434-453.


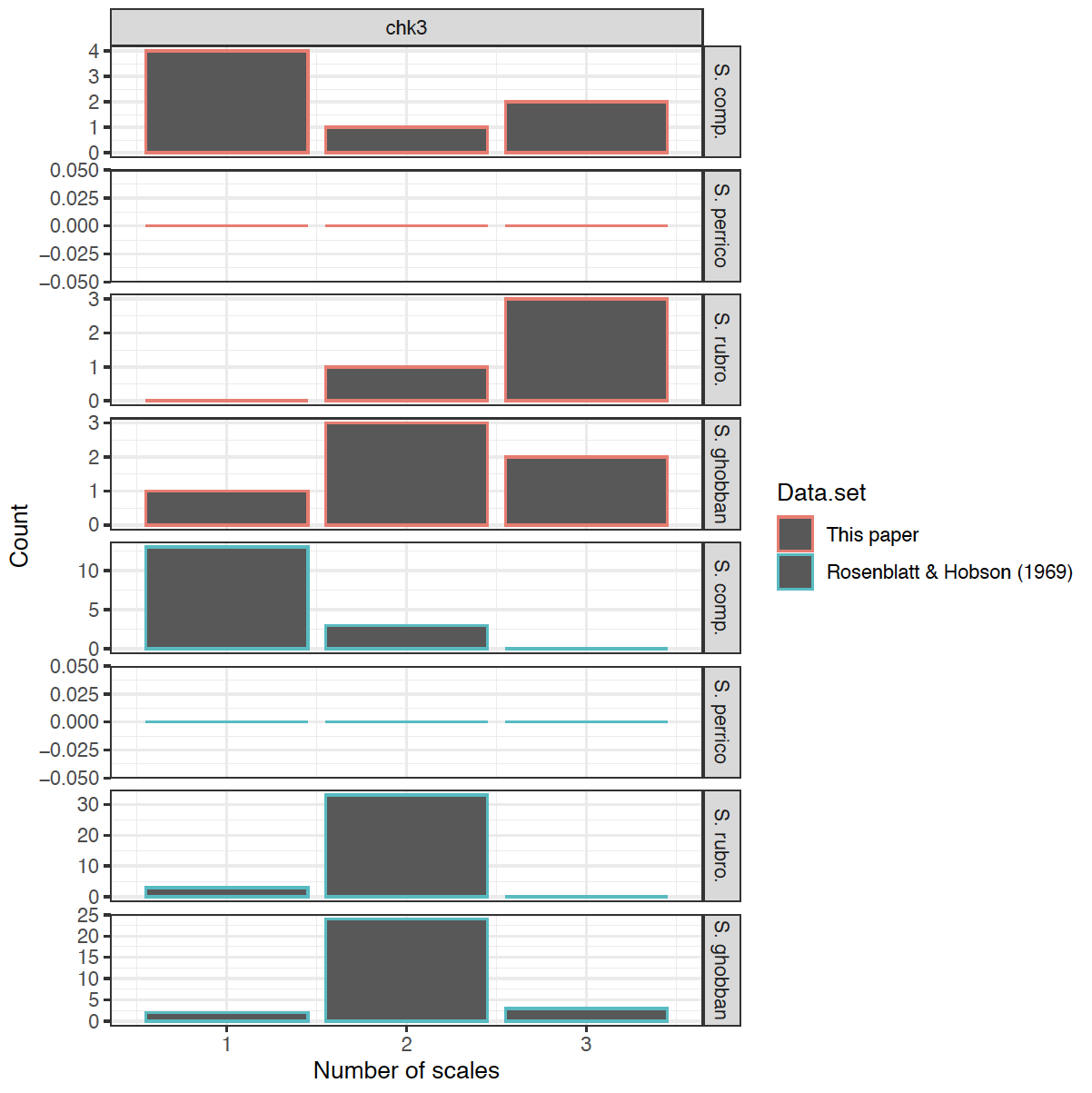


Figure S5b. Meristic data. Frequency distributions for scale counts in cheek scale row 3 (chk3). *Scarus perrico* does not have a 3^rd^ cheek scale row, represented by a lack of counts in the two plots. Upper panels with bars with red outline are data from samples collected in the Perlas Islands. Lower panels with bars and blue outline are data from Rosenblatt, R. H., and E. S. Hobson. 1969. Parrotfishes (Scaridae) of the eastern Pacific, with a generic rearrangement of the Scarinae. Copeia 434-453.


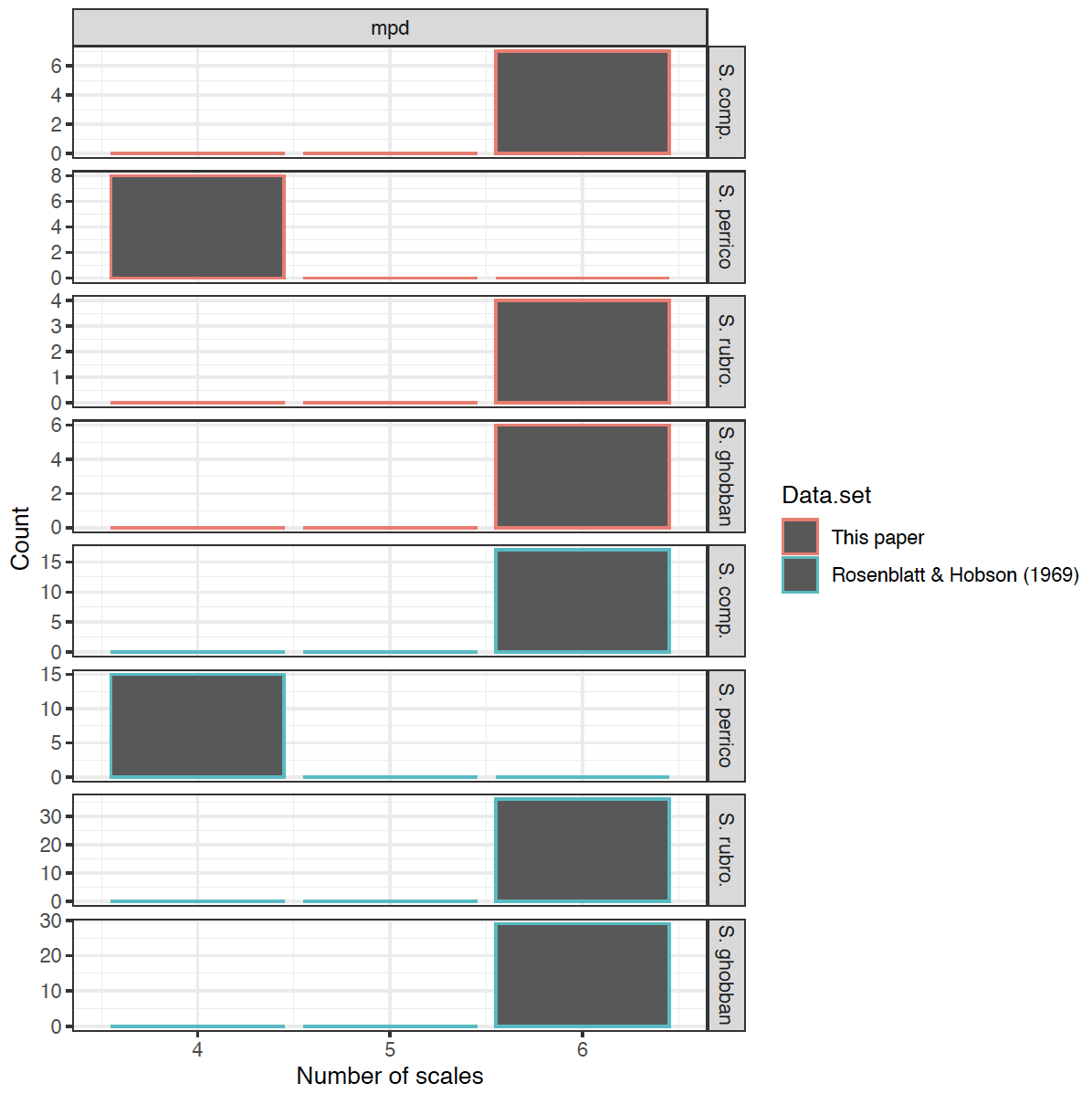


Figure S5c. Meristic data. Frequency distributions of median predorsal scale counts. Upper panels with bars with red outline are data from samples collected in the Perlas Islands. Lower panels with bars and blue outline are data from Rosenblatt, R. H., and E. S. Hobson. 1969. Parrotfishes (Scaridae) of the eastern Pacific, with a generic rearrangement of the Scarinae. Copeia 434-453.


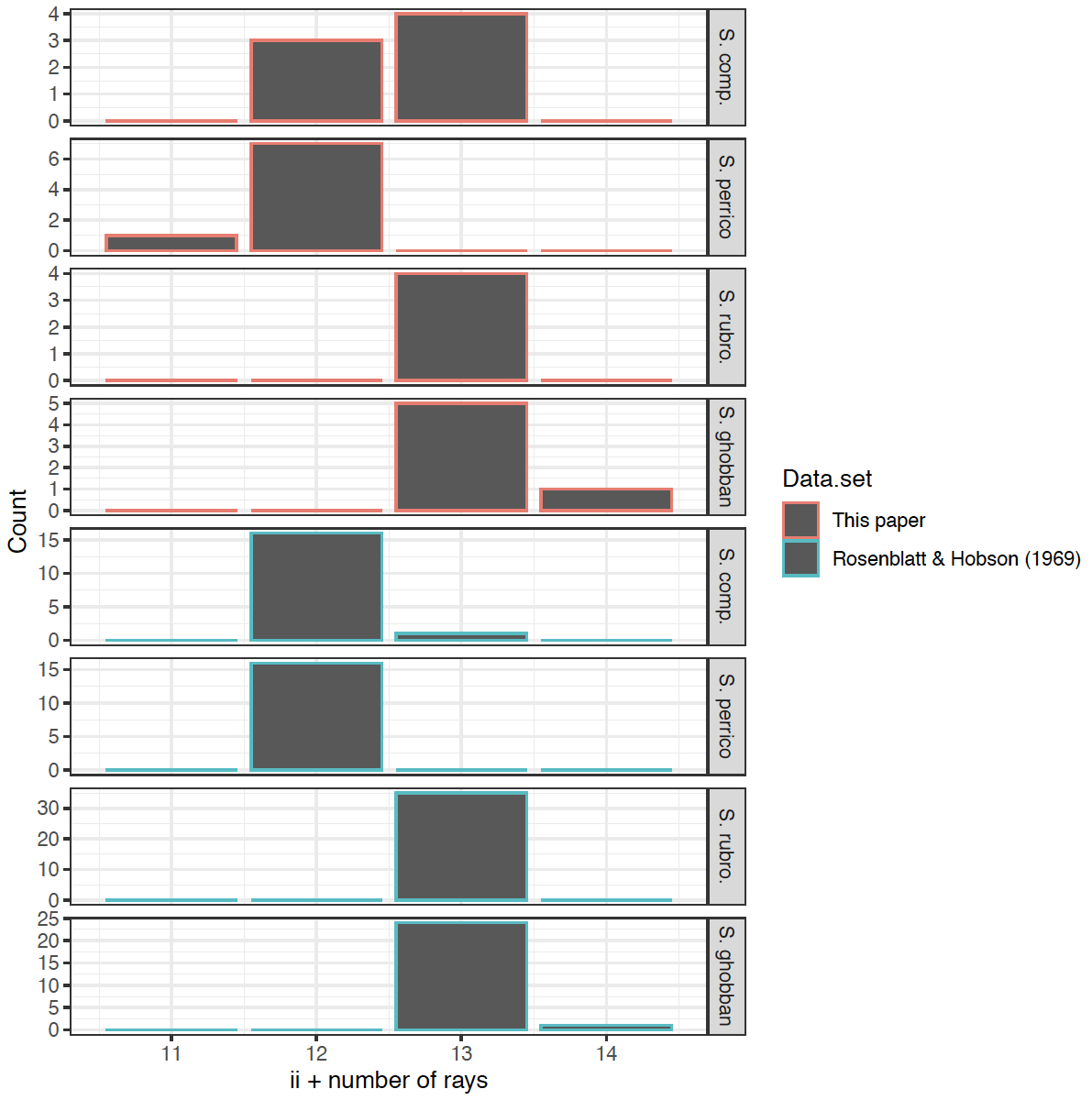


Figure S5d. Meristic data. Frequency distributions of ray counts in the pectoral fins. All species have two soft rays (ii) and a variable hard ray count (x axis). Upper panels with bars with red outline are data from samples collected in the Perlas Islands. Lower panels with bars and blue outline are data from Rosenblatt, R. H., and E. S. Hobson. 1969. Parrotfishes (Scaridae) of the eastern Pacific, with a generic rearrangement of the Scarinae. Copeia 434-453.

Figure S6a. Colour variation and admixture proportions (Q) among parental species and hybrids. Left columns are initial phases, and right columns are terminal phases. Photos in first two rows are the two parental species, and photos in the last row are ***S. perrico* × *S. ghobban* hybrids**. Phenotype, species specific Q values from K= 3 STRUCTURE model (Figure 2), sampling locality and catalogue # are listed below each photo.

Figure S6b. Colour variation and admixture proportions (Q) among parental species and hybrids. Left columns are initial phases, and right columns are terminal phases. Photos in first two rows are the two parental species, and photos in the last row are ***S. perrico* × *S. rubroviolaceus* hybrids**. Phenotype, species specific Q values from K= 3 STRUCTURE model (Figure 2), sampling locality and catalogue # are listed below each photo.

Figure S6c. Colour variation and admixture proportions (Q) among parental species and hybrids. Left columns are initial phases, and right columns are terminal phases. Photos in first two rows are the two parental species, and photos in the last row are ***S. ghobban* × *S. rubroviolaceus*** hybrids. Phenotype, species specific Q values from K= 3 STRUCTURE model (Figure 2), sampling locality and catalogue # are listed below each photo.
