# Supplementary material for: The origin of the parrotfish species Scarus compressus in the Tropical Eastern Pacific: region-wide hybridization between ancient species pairs
Source: BMC Ecol Evol. 2021 Jan 21;21:7. doi: 10.1186/s12862-020-01731-3 (PMC7853319; doi:10.1186/s12862-020-01731-3)
Supplement: Supplementary file 2 — Additional file 2: Table S1. Molecular diversity among species and localities. [file 12862_2020_1731_MOESM2_ESM.docx]

**Supplementary Table S1.** Additional file 2. Molecular diversity summary statistics for by gene, species, and locality.

|  |  | *S. compressus* | | | *S. ghobban* | | | *S. perrico* | | | *S. rubroviolaceus* | | |
| --- | --- | --- | --- | --- | --- | --- | --- | --- | --- | --- | --- | --- | --- |
|  |  | La Ventana | Perlas | Pixvae | La Ventana | Perlas | Pixvae | La Ventana | Perlas | Pixvae | La Ventana | Perlas | Pixvae |
| π | mtCR | 0.0761 | 0.0184 | 0.1137 | 0.0429 | 0.0775 | 0.0261 | 0.0153 | 0.0092 | 0.0104 | 0.0130 | 0.0030 | 0.0073 |
|  | bmp4 | 0.0067 | 0.0091 | 0.0079 | 0.0016 | 0.0034 | 0.0000 | 0.0008 | 0.0004 | 0.0000 | 0.0058 | 0.0029 | 0.0008 |
|  | Dlx2 | 0.0142 | 0.0106 | 0.0141 | 0.0046 | 0.0054 | 0.0053 | 0.0025 | 0.0000 | 0.0003 | 0.0090 | 0.0089 | 0.0000 |
|  | rag2 | 0.0137 | 0.0076 | 0.0131 | 0.0052 | 0.0067 | 0.0029 | 0.0035 | 0.0005 | 0.0006 | 0.0115 | 0.0094 | 0.0048 |
|  | Tmo4c4 | 0.0058 | 0.0039 | 0.0023 | 0.0016 | 0.0053 | 0.0003 | 0.0014 | 0.0000 | 0.0009 | 0.0049 | 0.0035 | 0.0013 |
|  |  |  |  |  |  |  |  |  |  |  |  |  |  |
| S | mtCR | 71 | 17 | 90 | 90 | 77 | 22 | 58 | 11 | 36 | 65 | 1 | 7 |
|  | bmp4 | 6 | 4 | 4 | 3 | 4 | 0 | 2 | 1 | 0 | 6 | 4 | 2 |
|  | Dlx2 | 14 | 8 | 13 | 10 | 8 | 5 | 9 | 0 | 1 | 12 | 11 | 0 |
|  | rag2 | 14 | 6 | 14 | 12 | 13 | 3 | 12 | 2 | 1 | 15 | 12 | 6 |
|  | Tmo4c4 | 8 | 3 | 7 | 6 | 6 | 1 | 6 | 0 | 3 | 7 | 6 | 3 |
|  |  |  |  |  |  |  |  |  |  |  |  |  |  |
| θ | mtCR | 18.4204 | 7.4453 | 26.1662 | 22.7178 | 25.4977 | 6.6300 | 14.2910 | 3.7556 | 9.7539 | 15.5705 | 1.0000 | 1.9457 |
|  | bmp4 | 1.3336 | 1.2890 | 0.9646 | 0.7945 | 1.0838 | 0.0000 | 0.5486 | 0.2678 | 0.0000 | 1.4280 | 1.1832 | 0.5486 |
|  | Dlx2 | 3.0359 | 2.5156 | 3.1350 | 2.3372 | 1.9712 | 1.2849 | 2.3835 | 0.0000 | 0.2678 | 2.9348 | 3.1981 | 0.0000 |
|  | rag2 | 3.1117 | 1.9868 | 3.3761 | 2.8561 | 3.4428 | 0.8230 | 3.2513 | 0.5297 | 0.2621 | 3.3799 | 3.2513 | 1.4674 |
|  | Tmo4c4 | 1.5810 | 0.9934 | 1.5361 | 1.6067 | 1.5567 | 0.3014 | 1.6067 | 0.0000 | 0.7945 | 1.6881 | 1.6912 | 0.8034 |
