# Supplementary material for: The origin of the parrotfish species Scarus compressus in the Tropical Eastern Pacific: region-wide hybridization between ancient species pairs
Source: BMC Ecol Evol. 2021 Jan 21;21:7. doi: 10.1186/s12862-020-01731-3 (PMC7853319; doi:10.1186/s12862-020-01731-3)
Supplement: Supplementary file 3 — Additional file 3: Table S2. Tests of linkage disequilibrium (LD) among sites for each species. [file 12862_2020_1731_MOESM3_ESM.docx]

**Supplementary Table S2.** Additional file 3. Tests of linkage disequilibrium by species, locality, and locus pair. The q-value is the probability of a false positive at $\alpha$= 0.05. The lfdr is the local false discovery rate, and the sig. column indicates significance, or q < 0.05.

| Species | Locality | Locus#1 | Locus#2 | P-Value | q-value | lfdr | sig. |
| --- | --- | --- | --- | --- | --- | --- | --- |
| *Scarus compressus* | Baja | Dlx2 | rag2 | 0.0252 | 0.0622 | 0.3629 | ns |
|  |  | Dlx2 | Tmo4c4 | 0.0000 | 0.0000 | 0.0000 | * |
|  |  | rag2 | Tmo4c4 | 0.0000 | 0.0000 | 0.0000 | * |
|  |  | Dlx2 | bmp4 | 0.0107 | 0.0295 | 0.1932 | * |
|  |  | rag2 | bmp4 | 0.0095 | 0.0280 | 0.1780 | * |
|  |  | Tmo4c4 | bmp4 | 0.0000 | 0.0000 | 0.0000 | * |
|  | Perlas | Dlx2 | rag2 | 0.0982 | 0.1846 | 0.9986 | ns |
|  |  | Dlx2 | Tmo4c4 | no table |  |  |  |
|  |  | rag2 | Tmo4c4 | no table |  |  |  |
|  |  | Dlx2 | bmp4 | 1.0000 | 1.0000 | 1.0000 | ns |
|  |  | rag2 | bmp4 | no table |  |  |  |
|  |  | Tmo4c4 | bmp4 | no table |  |  |  |
|  | Pixvae | Dlx2 | rag2 | 0.0013 | 0.0056 | 0.0411 | * |
|  |  | Dlx2 | Tmo4c4 | 0.0053 | 0.0177 | 0.1154 | * |
|  |  | rag2 | Tmo4c4 | 0.0074 | 0.0232 | 0.1476 | * |
|  |  | Dlx2 | bmp4 | 0.0002 | 0.0007 | 0.0078 | * |
|  |  | rag2 | bmp4 | 0.0015 | 0.0058 | 0.0455 | * |
|  |  | Tmo4c4 | bmp4 | 0.0000 | 0.0000 | 0.0000 | * |
| *Scarus ghobban* | Baja | Dlx2 | rag2 | 0.3555 | 0.4641 | 1.0000 | ns |
|  |  | Dlx2 | Tmo4c4 | 0.3907 | 0.4963 | 1.0000 | ns |
|  |  | rag2 | Tmo4c4 | 0.3204 | 0.4302 | 1.0000 | ns |
|  |  | Dlx2 | bmp4 | 0.1292 | 0.2150 | 1.0000 | ns |
|  |  | rag2 | bmp4 | 0.0340 | 0.0783 | 0.4528 | ns |
|  |  | Tmo4c4 | bmp4 | 0.2116 | 0.3108 | 1.0000 | ns |
|  | Perlas | Dlx2 | rag2 | 0.2062 | 0.3108 | 1.0000 | ns |
|  |  | Dlx2 | Tmo4c4 | 1.0000 | 1.0000 | 1.0000 | ns |
|  |  | rag2 | Tmo4c4 | 0.6735 | 0.7914 | 1.0000 | ns |
|  |  | Dlx2 | bmp4 | 0.6565 | 0.7911 | 1.0000 | ns |
|  |  | rag2 | bmp4 | 0.2863 | 0.3958 | 1.0000 | ns |
|  |  | Tmo4c4 | bmp4 | 0.0589 | 0.1259 | 0.6817 | ns |

| Species | Locality | Locus#1 | Locus#2 | P-Value | q-value | lfdr | sig. |
| --- | --- | --- | --- | --- | --- | --- | --- |
| *Scarus ghobban* | Pixvae | Dlx2 | rag2 | 0.0350 | 0.0783 | 0.4630 | ns |
|  |  | Dlx2 | Tmo4c4 | 1.0000 | 1.0000 | 1.0000 | ns |
|  |  | rag2 | Tmo4c4 | 1.0000 | 1.0000 | 1.0000 | ns |
|  |  | Dlx2 | bmp4 | no table |  |  |  |
|  |  | rag2 | bmp4 | no table |  |  |  |
|  |  | Tmo4c4 | bmp4 | no table |  |  |  |
| *Scarus perrico* | Baja | Dlx2 | rag2 | 0.1277 | 0.2150 | 1.0000 | ns |
|  |  | Dlx2 | Tmo4c4 | 0.0837 | 0.1638 | 0.8854 | ns |
|  |  | rag2 | Tmo4c4 | 0.0188 | 0.0490 | 0.2922 | * |
|  |  | Dlx2 | bmp4 | 0.0643 | 0.1314 | 0.7273 | ns |
|  |  | rag2 | bmp4 | 0.2592 | 0.3691 | 1.0000 | ns |
|  |  | Tmo4c4 | bmp4 | 0.1119 | 0.2022 | 1.0000 | ns |
|  | Perlas | Dlx2 | rag2 | no table |  |  |  |
|  |  | Dlx2 | Tmo4c4 | no table |  |  |  |
|  |  | rag2 | Tmo4c4 | no table |  |  |  |
|  |  | Dlx2 | bmp4 | no table |  |  |  |
|  |  | rag2 | bmp4 | no table |  |  |  |
|  |  | Tmo4c4 | bmp4 | no table |  |  |  |
|  | Pixvae | Dlx2 | rag2 | 1.0000 | 1.0000 | 1.0000 | ns |
|  |  | Dlx2 | Tmo4c4 | 0.1327 | 0.2150 | 1.0000 | ns |
|  |  | rag2 | Tmo4c4 | 0.5433 | 0.6719 | 1.0000 | ns |
|  |  | Dlx2 | bmp4 | no table |  |  |  |
|  |  | rag2 | bmp4 | no table |  |  |  |
|  |  | Tmo4c4 | bmp4 | no table |  |  |  |
| *Scarus rubroviolaceus* | Baja | Dlx2 | rag2 | 0.0000 | 0.0000 | 0.0000 | * |
|  |  | Dlx2 | Tmo4c4 | 0.0000 | 0.0000 | 0.0000 | * |
|  |  | rag2 | Tmo4c4 | 0.0000 | 0.0000 | 0.0000 | * |
|  |  | Dlx2 | bmp4 | 0.0000 | 0.0000 | 0.0000 | * |
|  |  | rag2 | bmp4 | 0.0048 | 0.0173 | 0.1073 | * |
|  |  | Tmo4c4 | bmp4 | 0.0000 | 0.0000 | 0.0000 | * |
|  | Perlas | Dlx2 | rag2 | small n |  |  |  |
|  |  | Dlx2 | Tmo4c4 | small n |  |  |  |
|  |  | rag2 | Tmo4c4 | small n |  |  |  |
|  |  | Dlx2 | bmp4 | small n |  |  |  |
|  |  | rag2 | bmp4 | small n |  |  |  |
|  |  | Tmo4c4 | bmp4 | small n |  |  |  |

| Species | Locality | Locus#1 | Locus#2 | P-Value | q-value | lfdr | sig. |
| --- | --- | --- | --- | --- | --- | --- | --- |
| *Scarus rubroviolaceus* | Pixvae | Dlx2 | rag2 | no table |  |  |  |
|  |  | Dlx2 | Tmo4c4 | no table |  |  |  |
|  |  | rag2 | Tmo4c4 | 0.1476 | 0.2313 | 1.0000 | ns |
|  |  | Dlx2 | bmp4 | no table |  |  |  |
|  |  | rag2 | bmp4 | 1.0000 | 1.0000 | 1.0000 | ns |
|  |  | Tmo4c4 | bmp4 | 1.0000 | 1.0000 | 1.0000 | ns |
