# Supplementary material for: The origin of the parrotfish species Scarus compressus in the Tropical Eastern Pacific: region-wide hybridization between ancient species pairs
Source: BMC Ecol Evol. 2021 Jan 21;21:7. doi: 10.1186/s12862-020-01731-3 (PMC7853319; doi:10.1186/s12862-020-01731-3)
Supplement: Supplementary file 4 — Additional file 4: Table S3. Results from Evanno method to choose the best Structure model. [file 12862_2020_1731_MOESM4_ESM.docx]

**Supplementary Table S3**. Additional file 4. Results from Evanno method to evaluate STRUCTRE models, K = 1 – 6. Bold text indicates best fitting model based on maximal Delta K value. Each model was simulated 13 times.

| K | Mean LnP(K) | Stdev LnP(K) | Ln'(K) | \|Ln''(K)\| | Delta K |
| --- | --- | --- | --- | --- | --- |
| 1 | -3295 | 0.06 | NA | NA | NA |
| 2 | -2566 | 11.75 | 729.65 | 150.82 | 12.84 |
| **3** | **-1987** | **0.40** | **578.84** | **602.17** | **1510.27** |
| 4 | -2010 | 1.21 | -23.33 | 5.55 | 4.57 |
| 5 | -2039 | 1.06 | -28.88 | 8.68 | 8.20 |
| 6 | -2059 | 1.42 | -20.20 | NA | NA |
