# Supplementary material for: The origin of the parrotfish species Scarus compressus in the Tropical Eastern Pacific: region-wide hybridization between ancient species pairs
Source: BMC Ecol Evol. 2021 Jan 21;21:7. doi: 10.1186/s12862-020-01731-3 (PMC7853319; doi:10.1186/s12862-020-01731-3)
Supplement: Supplementary file 5 — Additional file 5: Table S4. Type I and II error in simulations. [file 12862_2020_1731_MOESM5_ESM.docx]

**Supplementary Table S4.** Additional file 5. Type I and II error estimation from NewHybrids simulations for three crosses. Critical posterior probability threshold = 0.85.

| A. *S. perrico × S. rubroviolaceus* | | | | | | | | | | | | |
| --- | --- | --- | --- | --- | --- | --- | --- | --- | --- | --- | --- | --- |
|  | Calls within classes | | | Distribution of calls across classes | | | | | | | false positive | false negative |
|  | Class | Total called | Total called correctly | BC_PopA | BC_PopB | F1 | F2 | Pure PopA | Pure Pop B | Total simulated | Type I error | Type II error |
|  | BC_PopA | 6 | 0 | 0 | 0 | 3 | 0 | 3 | 0 | 50 | na | na |
|  | BC_PopB | 8 | 0 | 0 | 0 | 2 | 0 | 0 | 6 | 50 | na | na |
|  | F1 | 125 | 125 | 0 | 0 | 125 | 0 | 0 | 0 | 125 | 0.053 | 0 |
|  | F2 | 22 | 19 | 0 | 0 | 2 | 19 | 0 | 1 | 50 | 0 | 0.136 |
|  | Pure_PopA | 225 | 225 | 0 | 0 | 0 | 0 | 225 | 0 | 225 | 0.013 | 0 |
|  | Pure_PopB | 305 | 305 | 0 | 0 | 0 | 0 | 0 | 305 | 305 | 0.022 | 0 |
|  | Grand Total | 691 | 674 | 0 | 0 | 132 | 19 | 228 | 312 | 805 |  |  |
|  |  |  |  |  |  |  |  |  |  |  |  |  |
| B. *S. perrico × S. ghobban* | | | | | | | | | | | | |
|  | Calls within classes | | | Distribution of calls across classes | | | | | | | false positive | false negative |
|  | Class | Total called | Total called correctly | BC_PopA | BC_PopB | F1 | F2 | Pure PopA | Pure Pop B | Total simulated | Type I error | Type II error |
|  | BC_PopA | 14 | 0 | 0 | 0 | 1 | 1 | 12 | 0 | 50 | na | na |
|  | BC_PopB | 15 | 0 | 0 | 0 | 3 | 4 | 0 | 8 | 50 | na | na |
|  | F1 | 121 | 121 | 0 | 0 | 121 | 0 | 0 | 0 | 125 | 0.062 | 0 |
|  | F2 | 22 | 18 | 0 | 0 | 4 | 18 | 0 | 0 | 50 | 0.218 | 0.182 |
|  | Pure_PopA | 304 | 304 | 0 | 0 | 0 | 0 | 304 | 0 | 305 | 0.038 | 0 |
|  | Pure_PopB | 237 | 237 | 0 | 0 | 0 | 0 | 0 | 237 | 240 | 0.033 | 0 |
|  | Grand Total | 713 | 680 | 0 | 0 | 129 | 23 | 316 | 245 | 820 |  |  |

| C. S. ghobban *×* S. rubroviolaceus | | | | | | | | | | | | |
| --- | --- | --- | --- | --- | --- | --- | --- | --- | --- | --- | --- | --- |
|  | Calls within classes | | | Distribution of calls across classes | | | | | | | false positive | false negative |
|  | Class | Total called | Total called correctly | BC_PopA | BC_PopB | F1 | F2 | Pure PopA | Pure Pop B | Total simulated | Type I error | Type II error |
|  | BC_PopA | 8 | 3 | 3 | 0 | 0 | 0 | 5 | 0 | 25 | 0 | 0.625 |
|  | BC_PopB | 5 | 0 | 0 | 0 | 1 | 0 | 0 | 4 | 25 | na | na |
|  | F1 | 48 | 48 | 0 | 0 | 48 | 0 | 0 | 0 | 50 | 0.094 | 0 |
|  | F2 | 11 | 5 | 0 | 0 | 4 | 5 | 0 | 2 | 25 | 0 | 0.545 |
|  | Pure_PopA | 225 | 225 | 0 | 0 | 0 | 0 | 225 | 0 | 225 | 0.022 | 0 |
|  | Pure_PopB | 237 | 237 | 0 | 0 | 0 | 0 | 0 | 237 | 240 | 0.025 | 0 |
|  | Grand Total | 534 | 518 | 3 | 0 | 53 | 5 | 230 | 243 | 590 |  |  |
