# Supplementary material for: The origin of the parrotfish species Scarus compressus in the Tropical Eastern Pacific: region-wide hybridization between ancient species pairs
Source: BMC Ecol Evol. 2021 Jan 21;21:7. doi: 10.1186/s12862-020-01731-3 (PMC7853319; doi:10.1186/s12862-020-01731-3)
Supplement: Supplementary file 6 — Additional file 6: Table S5. Coalescence estimates based on Migrate Model 6 $\theta$θ estimates. [file 12862_2020_1731_MOESM6_ESM.docx]

**Supplementary Table S5**, Additional file 6. Estimates of the ratio of splitting time to effective population size (N_e_) for three parrotfish species: $\frac{t}{N_{e}}$ where *t* is measured in generations. Ratios are estimated based on the splitting time of the ancestors of *S. ghobban* and *S. rubrpviolaceus*= 4.36 Ma and the ancestor of these two species and the lineage that gave rise to *S. perrico*= 6.60 Ma (see Fig. 1). Effective population size estimates for each species use $\theta$ values from the best fitting Migrate model (Model 6, Table 2) and a mutation estimate for *mtCR* obtained by aligning a *Scarus hoefleri* sequence (GenBank accession #JX026563.1) to *S. perrico* sequences generated in this paper and estimating the number of substitutions between species (Ks = 26) along 375 bp. The splitting time for these two species is assumed to = 3 Ma and caused by the rise of the Isthmus of Panama. This gives mutation rate (u) of 1.16 x 10^-8^ per site per year, which was used to convert $\theta$ to *N_e_* with the formula: $N_{e}=\frac{\theta\times uS}{4\times u\times G}$ . Here, *uS* = the *mtCR* mutation scalar estimated by the Migrate model (3.5464), *u*= the mutation rate per site per year, and *G* = the generation time, assumed to be =5 years for all species.

| Species | $\theta$ | *N_e_* | $\frac{t}{N_{e}}$ (4.36 Ma) | $\frac{t}{N_{e}}$ (6.60 Ma) |
| --- | --- | --- | --- | --- |
| *Scarus ghobban* | 0.01203 | 184599 | 5 | 7 |
| *Scarus rubroviolaceus* | 0.00183 | 28081 | 31 | 47 |
| *Scarus perrico* | 0.00344 | 52787 | 17 | 25 |
