# Supplementary material for: The origin of the parrotfish species Scarus compressus in the Tropical Eastern Pacific: region-wide hybridization between ancient species pairs
Source: BMC Ecol Evol. 2021 Jan 21;21:7. doi: 10.1186/s12862-020-01731-3 (PMC7853319; doi:10.1186/s12862-020-01731-3)
Supplement: Supplementary file 7 — Additional file 7: Table S6. Count data for species, sites, and localities. [file 12862_2020_1731_MOESM7_ESM.docx]

**Supplementary Table S6.** Additional File 9. Ecological count data by site and locality. Species codes: *S.c.* = *Scarus compressus*, *S.g.* = *Scarus ghobban*, *S.p*. = *Scarus perrico*, *S.r.* = *Scarus rubroviolaceus*. The last column (% *S.c*.) is the % total that is the hybrid species, *Scarus compressus*. Counts include juveniles, initial phases (IP), and terminal phases (TP).

|  |  | Species | | | |  |  |
| --- | --- | --- | --- | --- | --- | --- | --- |
| Locality | Site | *S.c.* | *S.g.* | *S.p.* | *S.r.* | Total | % *S.c.* |
| La Ventana | Barco Varado | 0 | 36 | 13 | 0 | 49 | 0.00 |
| La Ventana | Cana Chica | 6 | 32 | 4 | 15 | 57 | 10.53 |
| La Ventana | Carriero | 0 | 12 | 4 | 52 | 68 | 0.00 |
| La Ventana | Cueva de Leon | 11 | 8 | 13 | 5 | 37 | 29.73 |
| La Ventana | El Mostrador | 9 | 2 | 23 | 21 | 55 | 16.36 |
| La Ventana | Ensenada de Muertos | 11 | 34 | 41 | 60 | 146 | 7.53 |
| La Ventana | Hornito | 4 | 20 | 19 | 68 | 111 | 3.60 |
| La Ventana | Hotel | 6 | 1 | 0 | 2 | 9 | 66.67 |
| La Ventana | Megana | 2 | 25 | 33 | 3 | 63 | 3.17 |
| La Ventana | Piedra Blanca | 12 | 29 | 44 | 41 | 126 | 9.52 |
| La Ventana | Punta Gorda S | 2 | 25 | 33 | 3 | 63 | 3.17 |
| La Ventana | Punta Gorda N | 2 | 45 | 25 | 0 | 72 | 2.78 |
| La Ventana | Punta Norte 1 | 6 | 8 | 16 | 10 | 40 | 15.00 |
| La Ventana | Punta Norte 2 | 2 | 8 | 2 | 1 | 13 | 15.38 |
| La Ventana | Punta Perrico | 10 | 24 | 103 | 52 | 189 | 5.29 |
| La Ventana | SW Point | 4 | 0 | 1 | 10 | 15 | 26.67 |
| La Ventana | Tecate | 7 | 7 | 10 | 3 | 27 | 25.93 |
| Perlas Islands | Contadoral NW | 0 | 1280 | 7 | 0 | 1287 | 0.00 |
| Perlas Islands | Hicaco Island | 9 | 0 | 29 | 51 | 89 | 10.11 |
| Perlas Islands | Isla Chitre W | 5 | 26 | 99 | 0 | 130 | 3.85 |
| Perlas Islands | Isla Marin | 4 | 7 | 159 | 32 | 202 | 1.98 |
| Perlas Islands | No Name 1 | 7 | 14 | 53 | 25 | 99 | 7.07 |
| Perlas Islands | No Name 2 | 12 | 0 | 20 | 8 | 40 | 30.00 |
| Perlas Islands | Pacheca East | 2 | 231 | 56 | 1 | 290 | 0.69 |
| Perlas Islands | Pacheca NE | 24 | 218 | 138 | 3 | 383 | 6.27 |
| Perlas Islands | Pachecquilla E | 3 | 25 | 20 | 0 | 48 | 6.25 |
| Perlas Islands | Pachequilla N | 1 | 21 | 9 | 0 | 31 | 3.23 |
| Perlas Islands | Pecheca SW | 0 | 165 | 40 | 0 | 205 | 0.00 |
| Perlas Islands | Pedro Gonzalez 15 | 4 | 49 | 63 | 2 | 118 | 3.39 |
| Perlas Islands | Pedro Gonzalez 16 | 5 | 5 | 115 | 2 | 127 | 3.94 |
| Perlas Islands | Pedro Gonzalez 17 | 6 | 15 | 55 | 1 | 77 | 7.79 |
| Perlas Islands | Saboga Islets | 1 | 136 | 15 | 0 | 152 | 0.66 |
| Perlas Islands | Saboga NW | 18 | 60 | 210 | 2 | 290 | 6.21 |
| Perlas Islands | Saboga Reef | 0 | 1754 | 12 | 0 | 1766 | 0.00 |
| Perlas Islands | Senorita Island | 1 | 71 | 48 | 2 | 122 | 0.82 |
| Perlas Islands | Site 10 | 2 | 68 | 37 | 0 | 107 | 1.87 |
| Perlas Islands | Site 11 | 5 | 103 | 32 | 0 | 140 | 3.57 |
| Perlas Islands | Soboga E | 5 | 26 | 99 | 0 | 130 | 3.85 |
| Perlas Islands | Taboquilla SW | 1 | 1 | 103 | 0 | 105 | 0.95 |
| Pixvae | 1 | 3 | 298 | 33 | 22 | 356 | 0.84 |
| Pixvae | 2 | 1 | 65 | 44 | 10 | 120 | 0.83 |
| Pixvae | 3 | 0 | 5 | 16 | 44 | 65 | 0.00 |
| Pixvae | 4 | 1 | 5 | 31 | 22 | 59 | 1.69 |
| Pixvae | 5 | 1 | 70 | 10 | 135 | 216 | 0.46 |
| Pixvae | 6 | 4 | 70 | 59 | 74 | 207 | 1.93 |
| Pixvae | 7 | 2 | 122 | 61 | 44 | 229 | 0.87 |
| Pixvae | 8 | 3 | 10 | 35 | 207 | 255 | 1.18 |
| Pixvae | 9 | 5 | 59 | 51 | 46 | 161 | 3.11 |
| Pixvae | 10 | 1 | 48 | 26 | 31 | 106 | 0.94 |
| Pixvae | 11 | 3 | 33 | 32 | 19 | 87 | 3.45 |
| Pixvae | 12 | 6 | 72 | 118 | 7 | 203 | 2.96 |
| Pixvae | 13 | 0 | 84 | 0 | 16 | 100 | 0.00 |
| Pixvae | 14 | 9 | 43 | 91 | 16 | 159 | 5.66 |
| Pixvae | 15 | 8 | 68 | 78 | 20 | 174 | 4.60 |
| Pixvae | 16 | 4 | 35 | 15 | 26 | 80 | 5.00 |
|  | Grand totals | 260 | 5678 | 2503 | 1214 | 9655 | 2.69 |
