# Supplementary material for: The origin of the parrotfish species Scarus compressus in the Tropical Eastern Pacific: region-wide hybridization between ancient species pairs
Source: BMC Ecol Evol. 2021 Jan 21;21:7. doi: 10.1186/s12862-020-01731-3 (PMC7853319; doi:10.1186/s12862-020-01731-3)
Supplement: Supplementary file 9 — Additional file 9. Observations of colour patterns and social behaviour in the field. [file 12862_2020_1731_MOESM9_ESM.docx]

**Additional file 8. Observations of colour patterns and social behaviour in the field**

*Scarus compressus* has two different colour phases, a brightly coloured terminal phase (TP) comprising large males, and a more dully coloured initial phase (IP). Initial phase fish usually were distinctly smaller than TPs in the same local population, but in Baja California at Cabo Pulmo National Park, a well-protected marine reserve, we saw large IPs almost as large as very large TPs (see *Scarus* hybrid movie, Additional file 7). The IP phase was quite variable in colour, much more variable than the IP patterns of the other three species. Some IP *S. compressus* had patterns that resemble that of TPs, while the patterns of others resembled that of an IP of one of the other three species. In the latter cases the difference between the colouration of IP *S. compressus* and that of another species ranged from being quite obvious to very subtle, with the latter including small differences in head shape and touches of colour to the beak or fin edges. *Scarus compressus* was typically observed singly, with TPs usually solitarily, and IPs either solitary, or, quite commonly, as individual members of harem groups of *S. rubroviolaceus* (see below). On the other hand, on two occasions (one at La Ventana, and one in Pixvae) we observed *S. compressus* in “harem groups” that consisted of a single TP and a single IP individual. Mulitiple members of both colour phases sometimes also joined large multispecies feeding schools of dozens to scores of adults of varying proportions of the other three species (see *Scarus* hybrid movie, Additional file 2), and we observed schools that included adults of all four species roaming actively across hard reef habitats at both sites in Panama and in Baja California. *S. compressus* is usually seen feeding on hard reef substrata, although it also feeds on sedimentary substrata.

*Scarus ghobban* was typically seen in small, loosely organized aggregations that move slowly across the substratum in sandy habitat around the bases of reefs. These aggregations included multiple individuals of both colour phases: brightly coloured TP males and more dully coloured IP individuals, which may include members of both sexes (Table 1). Individuals of this species also join multispecies feeding schools. *S. ghobban* usually is seen feeding on sedimentary substrata around the fringes of reefs.

*Scarus perrico* [see Robertson and Allen (2015) for additional colour variation] did not have two well-defined colour phases and the colour pattern of small adults includes all the pattern elements of that of large adults. The most noticeable difference between large and small adults is that large fish have more strongly developed head colours and can develop a large “cephalic hump” on the top of the head and nape, immediately in front of a patch of enlarged predorsal scales. However, observations on a school of ~50 large adults in the Cabo Pulmo National Park (~ 90 km south of the Ventana study site) show that only some of the largest adults develop a pronounced cephalic hump. Further, very large individuals can have extremely large cephalic humps and simplified colouration with exaggerated features of the colouration of smaller adults [see (Robertson and Allen 2015)]. Since we did not collect any such very large fish it is unclear whether or not there is any degree of sexual dimorphism in this species. In comparison to the other species *S. perrico* showed a much stronger tendency to form aggregations and both juveniles and adults of all sizes typically move around and feed in cohesive schools of tens to dozens of individuals. As with the other species *S. perrico* also participates in multispecies *Scarus* feeding schools. This species is more strongly associated with large areas of continuous reef substrata than are the other three species.

*Scarus rubroviolaceus* also had two well-defined colour phases, with the largest fish comprising TP males, and smaller IP fish including both sexes. At our study sites *S. rubroviolaceus* typically was seen in small harem groups composed of a large TP and several smaller IPs, with the TP fish aggressively defending the area within which the group feeds against approaches by conspecifics. Large IPs in such groups also are involved in aggressive interactions with approaching IP conspecifics. As noted above, individual *S. compressus* IPs that resemble IP *S rubroviolaceus* in colour often participated in such social groups in both Mexico and Panama.

Reference

Robertson, D. R., and G. R. Allen. 2015. Shorefishes of the Tropical Eastern Pacific: online information system. Version 2.0 Smithsonian Tropical Research Institute, Balboa, Panamá.
