# Supplementary material for: The origin of the parrotfish species Scarus compressus in the Tropical Eastern Pacific: region-wide hybridization between ancient species pairs
Source: BMC Ecol Evol. 2021 Jan 21;21:7. doi: 10.1186/s12862-020-01731-3 (PMC7853319; doi:10.1186/s12862-020-01731-3)
Supplement: Supplementary file 10 — Additional file 10. Expected heterozygosity with mixed intra- and interspecific mating. [file 12862_2020_1731_MOESM10_ESM.docx]

**Additional file 10. The expected heterozygosity with mixed intra- and interspecific mating**

If *S. compressus* is a fourth species and is therefore practicing some assortative mating, then the proportion of the species that is assortatively mating must be small relative to the proportion that is hybridizing with other species to be consistent with the high levels of multilocus heterozygosity found in our *S. compressus* samples: 90% of the *S. compressus* samples had four-locus heterozygosity. If we assume 4 equally common alleles across loci (~ 3-4 common alleles per locus were observed in our nuclear data, see Fig. 2b-e), after one generation of random mating among F1 hybrids the probability of heterozygosity across all loci can be expressed as:

$${P\left( 4 locus heterozygosity \right)= [(2 \times{0.25}^{2})*6]}^{4}=0.32$$

The probability of homozygosity at one or more loci is therefore:

$$P\left( \geq1 locus homozygosity \right)=1- P\left( 4 locus heterozygosity \right)=0.68$$

However, we observed a $P\left( \geq1 locus homozygosity \right)= 0.10$ in our data. In order to fit our data to a hybrid complex that includes a freely hybridizing fourth species such as *S. compressus*, 68% of the *S. compressus* individuals would be hybridizing with one of the other species in the complex, while the remaining 32% would be mating with conspecifics. Our ecological counts found the mean relative abundance of *S. compressus* was < 10% of the total count at all localities, and often only a few % of the total count (Fig 5a). These densities would present a paradox for the success and persistence of assortative mating and a fourth species.
