# Supplementary material for: The origin of the parrotfish species Scarus compressus in the Tropical Eastern Pacific: region-wide hybridization between ancient species pairs
Source: BMC Ecol Evol. 2021 Jan 21;21:7. doi: 10.1186/s12862-020-01731-3 (PMC7853319; doi:10.1186/s12862-020-01731-3)
Supplement: Supplementary file 11 — Additional file 11: Table S7. PCR primers and conditions. [file 12862_2020_1731_MOESM11_ESM.docx]

**Supplementary Table S7.** Additional File 10. PCR primers, annealing temperatures, and gene features of four nuclear markers and the mitochondrial control region.

| Gene | Primers | Sequence | Alignment length (bp) | Annealing temperature (°C) | Gene description and features |
| --- | --- | --- | --- | --- | --- |
| *rag2* | Rag2-38F  Rag2-535R | Forward: GAAAAGAGTGTTTGAAAATGA  Reverse: CATCGTGCTCCTGGGTGACAAAGT | 342 | 55 | Recombination activating gene-2, single exon, partial sequence |
| *Tmo4c4* | Tmo-f1-6  Tmo-r1-3 | Forward: GAAAAGAGTGTTTGAAAATGA  Reverse: CATCGTGCTCCTGGGTGACAAAGT | 419 | 55 | Similar to human titan protein, single protein, partial sequence |
| *Dlx2* | Dlx2-F760  Dlx2-R2 | Forward: GAAGAGAGYGAGCCAGAAATC  Reverse: AGTTTGCCAAAAACGACGAA | 320 | 55 | Homolog of the Drosophila  homeodomain transcription factor (*dll*). Partial sequence of Exon 2 & 3 and complete intervening intron |
| *bmp4* | Bmp4-2F34  Bmp4-2R375 | Forward: CACACCTCTTCGCTTCCTGT  Reverse: TGGTGCGGTGAAGTCTTGTT | 236 | 55 | Vertebrate homolog of *Drosophila* decapentaplegic (*dpp*). Partial sequence of Exon 4 |
| *mtCR* | mtCR-L15995  mtCR-H16498 | Forward: AATTCTCACCCCTAGCTCCCAAA  Reverse: CCTGAAGTAGGAACCAGATG | 375 | 52 | Mitochondrial control region, partial sequence |
