# Supplementary material for: The origin of the parrotfish species Scarus compressus in the Tropical Eastern Pacific: region-wide hybridization between ancient species pairs
Source: BMC Ecol Evol. 2021 Jan 21;21:7. doi: 10.1186/s12862-020-01731-3 (PMC7853319; doi:10.1186/s12862-020-01731-3)
Supplement: Supplementary file 12 — Additional file 12. The power of hybrid assignment. [file 12862_2020_1731_MOESM12_ESM.docx]

**Additional file 12. The power of hybrid assignment**

Power to correctly assign parental and hybrid classes depends on the number of unlinked loci in the marker panel and the differences in allele frequencies between the two parental populations at each locus (Anderson and Thompson 2002). To determine power of our nuclear data set we ran simulations using the Hybriddetective workflow (Wringe et al. 2017) in the R environment. This package estimates five components of power at a given posterior probability of assignment (Vähä and Primmer 2006), and we focused on three of these: i. efficiency - the number of individuals of known hybrid class correctly assigned over the total number of individuals known to belong to that class, i. accuracy - the number of individuals correctly assigned to a class over the total number of individuals assigned to that class, and iii. power - the product of efficiency and accuracy. In addition to these power metrics, we also calculated Type I and II error rates from the simulated data. Here, the probability of a Type I error (e.g. the false positive rate) was equal to the number of individuals that are incorrectly assigned to a given class divided by the total number of individuals that are assigned to that class. The probability of a Type II error (e.g. the false negative rate) was equal to the number of individuals of a given class that are incorrectly assigned to that class divided by the total number of individuals of that class. For each cross, we simulated five replicate populations using parental genotypes with Q-values ≥ 0.90 in from the K=3, Structure model. For each replicate population, we simulated the same number of parental genotypes as occurred in the complete data set and a distribution of hybrid genotypes that included 25 F1 hybrid genotypes, 10 F2 hybrid genotypes, and 10 hybrid backcross genotypes in each direction. We chose these hybrid proportions based on the relative abundance estimates of *S. compressus* at the three sites (2 – 14 %, Fig. 2a) and the logic that F2 hybrids and backcrosses should be numerically rarer than F1 if there are no large fitness advantages of later-generation hybrids relative to pure individuals and F1 hybrids.

References

Anderson, E. C., and E. A. Thompson. 2002. A model-based method for identifying species hybrids using multilocus genetic data. Genetics 160:1217-1229.

Vähä, J. P., and C. R. Primmer. 2006. Efficiency of model‐based Bayesian methods for detecting hybrid individuals under different hybridization scenarios and with different numbers of loci. Molecular Ecology 15:63-72.

Wringe, B. F., R. R. E. Stanley, N. W. Jeffery, E. C. Anderson, and I. R. Bradbury. 2017. hybriddetective: A workflow and package to facilitate the detection of hybridization using genomic data in r. Molecular Ecology Resources 17:e275-e284.
