# Supplementary material for: The origin of the parrotfish species Scarus compressus in the Tropical Eastern Pacific: region-wide hybridization between ancient species pairs
Source: BMC Ecol Evol. 2021 Jan 21;21:7. doi: 10.1186/s12862-020-01731-3 (PMC7853319; doi:10.1186/s12862-020-01731-3)
Supplement: Supplementary file 13 — Additional file 13. Testing Darwin’s corollary. [file 12862_2020_1731_MOESM13_ESM.docx]

**Additional file 13. Testing for rate asymmetry between mitochondrial and nuclear genes**

We first constructed a phylogenetic hypothesis for the 22 species of a monophyletic clade that includes the TEP *Scarus* species, using *Scarus xanthrops* as the outgroup. Our phylogeny was based on a concatenated alignment of five mt (*12S*, *16S*, *CO1*, *CytB*, *Control Region*) and six nuclear genes (*bmp4*, *Dlx2*, *Otx1*, *rag2*, *S7*, *Tmo4c4*) (Siqueira et al. 2016) kindly provided by A. C. Siqueira, which we edited to include the target species and supplemented with *S. perrico* sequences generated from this study. Briefly, we used the gene-specific branch lengths *L* from the two species of each potential crosses to their most recent common ancestor (MRCA) as estimates of the rate of molecular evolution. To quantify asymmetry in branch lengths we estimated the following parameters following the notation of (Brandvain et al. 2014). If C is the MRCA of hybridizing species A and B, then *L(m)_Ci_* represents the number of mitochondrial substitutions between C and species *i*. Similarly, *L(n)_Ci_* is the number of nuclear substitutions between C and i. An estimate of asymmetry in the two branch lengths (*L(m)_CA_* and *L(m)_CB_*) is provided by $v_{m}=\frac{{L(m)}_{CA}}{{L(m)}_{CA}+{L(m)}_{CB}}$ , where $v_{m}>0.5$ indicates accelerated mitochondrial evolution in species A compare to species B. Similarly, asymmetry in the two nuclear branch lengths is estimated by *v_n_* in the same way. Focusing on species A, an estimate of the difference in asymmetry between mitochondrial and nuclear branch lengths (i.e. acceleration in the mitochondrial rate compared to the nuclear rate) is given by $\delta_{A}=v_{m}-v_{n}$, where $\delta_{A}>0$ indicates species A has a faster evolution of the mitochondrial vs. nuclear gene. If $\delta_{A}<0$, species A has a slower rate of evolution of the mitochondrial vs. nuclear gene. This reasoning can be extended to multiple genes within each class (mt and nuclear) by summing the branch lengths for the L estimates within classes. By example, If DMIs involving nuclear and mitochondrial genes are leading to asymmetry in reciprocal crosses between species A and B and we do not observe mitochondrial lineage A in a sample of A $\times$ B hybrids, we expect $\delta_{A}>0$. To obtain a phylogenetic hypothesis among all species, we used our supplemented alignment to generate a maximum likelihood tree using RAxML-NG (Kozlovet al. 2019). Before running the tree, we first determined the most likely model of evolution for each of the 11 partitions (corresponding to each mt and nuclear gene) using the AIIC criterion in PartitionFinder 2 (Lanfear et al. 2017). We then used the best fitting partition scheme and RAxML and the scaled branch option (--brlen scaled) to find the most likely topology. We checked for convergence by using 50 different starting trees, which all arrived at the same ML score and topology. We used this topology to constrain an analysis of gene-specific branch lengths with the --evaluate option in RAxML by unlinking the branch lengths within the individual partitions (--brlen unlinked). The resulting Newick trees for each partition where converted to inter-node branch distances using the R libraries APE and Phytools, and$v$ and $\delta$ parameters (as described above) were calculated for each of the three species pairs.

References

Brandvain, Y., G. B. Pauly, M. R. May, and M. Turelli. 2014. Explaining Darwin’s corollary to Haldane’s rule: the role of mitonuclear interactions in asymmetric postzygotic isolation among toads. Genetics 197:743-747.

Kozlov, A. M., D. Darriba, T. Flouri, B. Morel, and A. Stamatakis. 2019. RAxML-NG: a fast, scalable and user-friendly tool for maximum likelihood phylogenetic inference. Bioinformatics 35:4453-4455.

Lanfear, R., P. B. Frandsen, A. M. Wright, T. Senfeld, and B. Calcott. 2017. PartitionFinder 2: new methods for selecting partitioned models of evolution for molecular and morphological phylogenetic analyses. Molecular Biology and Evolution 34:772-773.

Siqueira, A. C., L. G. R. Oliveira‐Santos, P. F. Cowman, and S. R. Floeter. 2016. Evolutionary processes underlying latitudinal differences in reef fish biodiversity. Global Ecology and Biogeography 25:1466-1476.
